# Transcriptomic and functional responses of the cystic fibrosis airway epithelium to CFTR modulator therapy

**DOI:** 10.1172/jci.insight.196018

**Published:** 2025-11-10

**Authors:** Eszter K. Vladar, Austin E. Gillen, Sangya Yadav, Mikayla R. Murphree, David Baraghoshi, J. Kirk Harris, Elmar Pruesse, Sierra S. Niemiec, Alexandra W.M. Wilson, Katherine B. Hisert, Stephen M. Humphries, Matthew Strand, David A. Lynch, Max A. Seibold, Daniel M. Beswick, Jennifer L. Taylor-Cousar

**Affiliations:** 1 Department of Medicine, University of Colorado, Aurora, Colorado, USA.; 2 Rocky Mountain Regional Veterans Affairs Medical Center, Aurora, Colorado, USA.; 3 Division of Biostatistics, National Jewish Health, Denver, Colorado, USA.; 4 Departments of Pediatrics, University of Colorado, Aurora, Colorado, USA.; 5 Center for Genes, Environment, and Health, and; 6 Department of Pediatrics, National Jewish Health, Denver, Colorado, USA.; 7 Department of Biostatistics & Informatics, University of Colorado, Aurora, Colorado, USA.; 8 Clinical Research Services,; 9 Department of Medicine, and; 10 Department of Radiology, National Jewish Health, Denver, Colorado, USA.; 11 Department of Otolaryngology-Head and Neck Surgery, UCLA, Los Angeles, California, USA.

**Keywords:** Inflammation, Pulmonology, Cellular immune response, Monogenic diseases, Transcriptomics

## Abstract

Elexacaftor/tezacaftor/ivacaftor (ETI) cystic fibrosis transmembrane conductance regulator (CFTR) modulator therapy has led to rapid and substantial improvements in cystic fibrosis (CF) airway disease. Underlying molecular and cellular mechanisms, long-term efficacy, and ability to reverse airway epithelial remodeling in established disease remain unclear. Longitudinal nasal brushes from an adult CF cohort were used to evaluate gene expression, cellular composition, stem cell function, and microbiome changes at baseline and at 6 months and 2 years after ETI. The baseline to 6 month span showed a massive downregulation of extensive neutrophilic inflammatory gene expression programs that correlated with increased pulmonary function and decreased sinusitis. Primary airway epithelial stem cell cultures from matched donor samples showed partially improved differentiation and barrier capacity at 6 months. Although clinical outcomes remained stable during the 6 month to 2 year span, transcriptional changes revealed a resurgence of baseline inflammatory programs. The time course of gene expression was consistent with ongoing normalization of epithelial remodeling. Relative abundance of *Pseudomonas* also decreased during the time course. These data suggest that ETI rectifies inflammation, epithelial remodeling, and bacterial infection in the airways, but resurgence of inflammatory gene expression may indicate ongoing inflammation, potentially presaging disease progression with long-term therapy.

## Abstract 2

Longitudinal profiling of the cystic fibrosis airway epithelium reveals partially reduced inflammation, infection, and normalization of epithelial function in response to CFTR modulator therapy.

## Introduction

Cystic fibrosis (CF) airway disease arises secondary to defects in the cystic fibrosis transmembrane conductance regulator (CFTR) ion channel that lead to dehydration of the airway surface and accumulation of thick, difficult to clear mucus that acts as a nidus for polymicrobial infections (1–3). Cycles of chronic infection, inflammation, and injury lead to structural and functional defects, termed remodeling, in the airway epithelium. Remodeled epithelia are characterized by abnormal basal stem cells, excess mucous and squamous cells, fewer ciliated cells, and infiltrating immune cell populations, leading to defects in mucociliary, barrier, and regenerative functions. Since its introduction in 2019, elexacaftor/tezacaftor/ivacaftor (ETI) has led to substantial improvements in symptoms and clinical measures of CF airway disease (4, 5). Most people with CF (pwCF) carry at least one copy of an ETI responsive CFTR variant, which has resulted in widespread use in countries where it is available. ETI facilitates the proper folding, apical transport, and activation of CFTR to restore epithelial ion transport, which improves mucociliary function and attenuates infection and inflammation (5). However, the underlying molecular and cellular mechanisms are incompletely understood and long-term efficacy and ability to halt or reverse epithelial remodeling in pwCF with established airway disease remain unclear.

Studies focused on the airway microbiome and host cellular and molecular changes in sputum and nasal brush biopsies indicate that infection and inflammation are rapidly and markedly reduced after initiating ETI in adults with established CF lung disease (6–13). Classic markers of CF airway inflammation such as IL-1β, IL-6, IL-8, TNF-α, and neutrophil elastase as well as neutrophil abundance in sputum were significantly decreased as early as 1 month and remained low when measured more than 1 year after ETI. Importantly, inflammatory markers remained higher than those detected in healthy individuals. These changes were mirrored by a decrease in but not eradication of *Pseudomonas aeruginosa*, a dominant CF airway pathogen in both sputum and sinonasal samples in older adults. Nasal epithelial transcriptomics comparing gene expression at baseline to 3–12 months after ETI produced only a modest number of changes, likely due to the variable timing of sample collection, although genes related to inflammation and mucin production were decreased (14). A separate study using primary cultured CF airway epithelial cells treated with ETI compounds in vitro also identified a few differentially expressed genes related to bacterial defense and cellular stress response and increased CFTR-dependent chloride transport (15).

The current study evaluated sinonasal airway mucosal gene expression, cellular composition, and microbiome changes in nasal brushes at baseline and at 6 months (6 mo.) and 2 years (2 yr.) after initiating ETI therapy in a previously described, richly clinically phenotyped adult CF cohort (16–23). Pulmonary function (measured as percent predicted forced expiratory volume in 1 second; ppFEV1), CF-related chronic rhinosinusitis (CRS), olfactory function, and patient-reported outcomes quantifying sinonasal quality of life, productivity loss, and health utility showed substantial improvements in lung disease and all measured aspects of sinus disease except for olfaction from baseline to 6 mo. and no significant cohort-level change from 6 mo. to 2 yr. follow-up. These results are highly consistent with multiple other studies reporting rapid and sustained improvements in lung and sinus disease.

Transcriptomic outcomes for baseline to 6 mo. after ETI therapy showed extensive and highly significant changes in the sinonasal epithelial and immune compartments, indicating broadly attenuated neutrophilic inflammation and improved epithelial remodeling. Downregulation of inflammatory gene expression programs, which included well-known CF airway molecular biomarkers, was associated with increased ppFEV1 and decreased CRS severity. Baseline to 6 mo. transcriptional changes were consistent with reduced neutrophil and macrophage and increased T cell recruitment to the sinonasal airway epithelium. Primary airway epithelial stem cells differentiated in vitro from matched donor samples showed mostly improved barrier function and ciliated cell formation at 6 mo. compared with baseline. Importantly, 6 mo. to 2 yr. transcriptional changes revealed a resurgence of baseline inflammatory programs, while clinical outcomes did not significantly change during this timeframe. Six mo. to 2 yr. gene expression changes reflected continued normalization of epithelial remodeling despite increased inflammatory gene expression. *Pseudomonas* spp. relative abundance also decreased during the time course. These data suggest that ETI reduces inflammatory airway disease and infection, but transcriptomic resurgence of inflammatory gene expression may indicate ongoing inflammation despite therapy.

## Results

### Extensive longitudinal transcriptional changes underlie the sinonasal mucosal response to initiating ETI in an adult cohort of pwCF.

**Figure 1:**
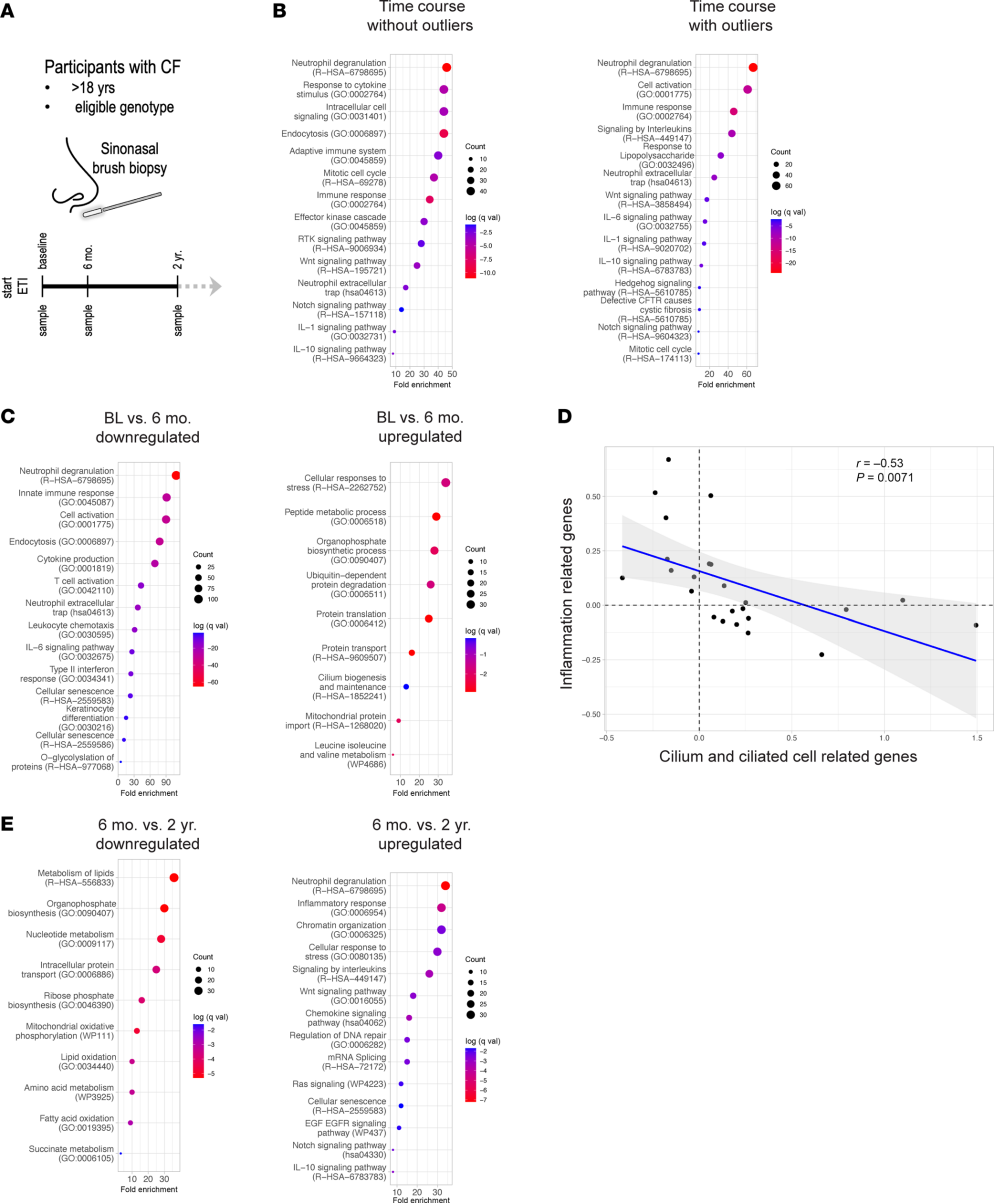
Sinonasal differential gene expression in response to ETI therapy. (**A**) Study schematic with time points. (**B**) Pathway enrichments for genes differentially expressed across the entire time course. (**C**) Pathway enrichments for up- and downregulated genes from baseline to 6 mo. (**D**) Plot of Pearson’s correlation of gene expression related to cilia and ciliated cells and airway epithelial inflammation. (**E**) Pathway enrichments for up- and downregulated genes from 6 mo. to 2 yr.

To delineate longitudinal changes in cellular programming and function within the sinonasal airway epithelium in response to ETI in a cohort of adult pwCF with established lung and sinus disease (16–23), participants were recruited for nasal brushing of the inferior turbinates at baseline and at 6 mo. and 2 yr. after initiating ETI therapy. Samples were obtained from a total of 30 participants, including 28, 24, and 23 participants at baseline, 6 mo., and 2 yr., respectively (Supplemental Table 1; supplemental material available online with this article; https://doi.org/10.1172/jci.insight.196018DS1), representing 21 complete time courses, 24 baseline–6 mo. pairs, 22 baseline–2 yr. pairs, and 21 6 mo.–2 yr. pairs. Cells obtained from the left nostril were banked for subsequent primary airway epithelial cell culture, while cells obtained from the right nostril were subjected to whole transcriptome RNA sequencing (Figure 1A). Principal component analysis (PCA) of the transcriptomic data identified two 2 yr. samples as potential outliers (Supplemental Figure 1); therefore, analyses were carried out both with and without these samples. Differential gene expression testing (adj. *P* < 0.05) across the experimental time points yielded a total of 919 genes including the outliers and 806 excluding the outliers (Supplemental Table 2). Including or excluding outliers, the most highly enriched molecular pathways were related to neutrophilic inflammation (*SERPINA1*, *IL1A*, *IL1RN*, *TLR4*, *PECAM1*, *NLRP3*, *FCGR2A*), cell cycle regulation (*AURKB*, *CENPF*, *FOXM1*), and cell signaling (*EREG*, *LRP5*, *NUMB*) (Figure 1B). Genes related to motile cilium biogenesis (*CEP55*, *CETN3*, *DNAH14*) and squamous/cornified epithelium (*IVL*, *SPRR3*, *KRT13*) were also significantly differentially expressed. Including outliers yielded additional immune function–related genes, including those responsive to viral infection (*CCL20*, *CXCR5*, *MX2*), suggesting that these 2 samples may represent a more highly inflammatory state. Of note, one of the participants reported flu-like symptoms at the time of sampling. Downstream analyses exclude outliers unless otherwise noted. One additional participant underwent lung transplantation between the 6 mo. and 2 yr. time points, so the 2 yr. time point for this participant was excluded from pulmonary function–related comparisons (Supplemental Table 1). In sum, time course gene expression signatures in this cohort capture a drastically changing molecular landscape related to inflammatory epithelial remodeling in the CF sinonasal airways in response to ETI.

### Sinonasal transcriptional responses from baseline to 6 mo. reflect decreased neutrophilic inflammation and improved epithelial remodeling.

We hypothesized that the rapid and marked improvement in CF airway disease upon initiating ETI is accompanied by changes in gene expression that reflect decreased inflammation and normalization of epithelial structure and function. Consistent with this hypothesis, we observed 865 downregulated genes (adj. *P* < 0.05) from baseline to 6 mo. that were dominated by those related to neutrophil degranulation, neutrophil extracellular trap formation, cytokine production and signaling, and chemotaxis, and included critical drivers of CF airway inflammation such as *CXCL8*, *CXCR1*, *CXCR2*, *TLR4*, *PTGS2*, and *IL1R2* (2). Genes related to cell senescence and senescence-associated secretory phenotype (24) (SASP; *FOS*, *CDKN2D*, *MAPK3*) were also markedly downregulated (Figure 1C and Supplemental Table 3). The 631 baseline to 6 mo. upregulated genes (adj. *P* < 0.05) were involved in various metabolic processes (*GLS2*, *DHDH*, *ACADL*), organelle biogenesis and function, including motile cilium biogenesis (*WDPCP*, *CEP57*, *BBS10*), and cell cycle control (*CDK4*, *ORC3*, *ANAPC16*) (Figure 1C and Supplemental Table 3). These data identify molecular factors underlying the widely reported attenuation of inflammatory drivers of disease after ETI therapy, and further indicate a shift toward normalization of airway epithelial structure and function. This finding was further supported by a significant negative correlation (*r* = –0.53, *P* = 0.0071) in expression between genes related to motile cilia and ciliated cells (25) and genes related to sinonasal airway epithelial inflammation (26) for the baseline to 6 mo. span (Figure 1D).

**Figure 2:**
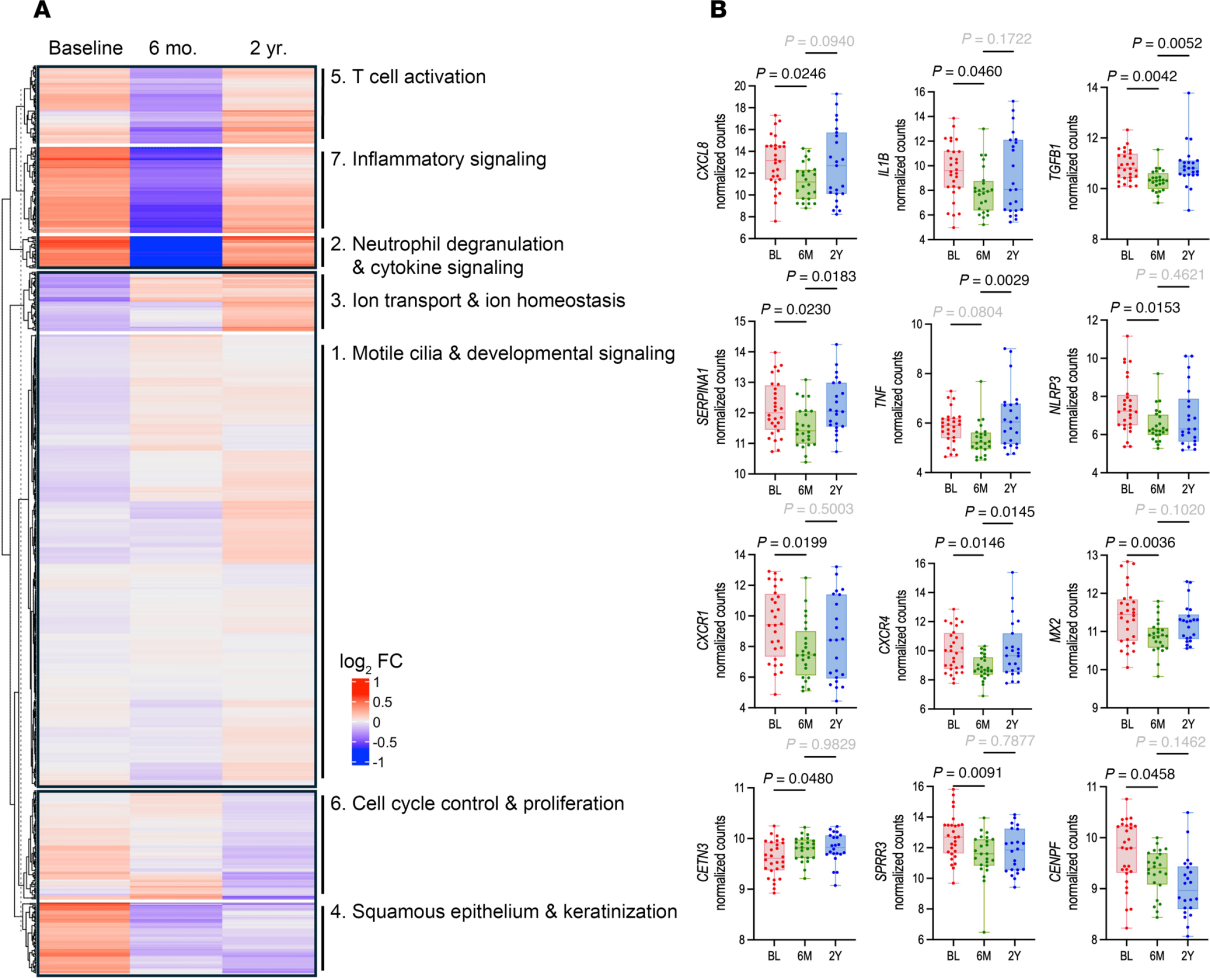
Decreased inflammatory gene expression reverses after long-term ETI therapy. (**A**) Hierarchical clustering of differentially expressed genes during the time course. Heatmap shows the mean-centered log_2_(fold change) in expression. (**B**) Box-and-whisker plots of select individual genes of interest related to immune and epithelial cell processes. Significance assessed by 1-way ANOVA with Dunnett’s post hoc test, 2 yr. transcriptional outliers excluded.

To delineate global cellular and molecular processes involved in the transcriptional response to ETI, hierarchical clustering based on shared patterns of expression was used to identify 7 gene clusters (Figure 2A and Supplemental Table 4). The 3 clusters related to inflammation exhibited strongly decreased expression from baseline to 6 mo. These clusters included genes related to neutrophil degranulation and cytokine signaling (Cluster 2: *FCGR2A*, *FGR*, *LCP2*, *CD53*, *CSF3R*), T cell activation (Cluster 5: *TNFSF8*, *IL1A*, *IL2RG*), and inflammatory signaling (Cluster 7: *NLRP3*, *NLRP6*, *TLR4*, *TLR8*). Three clusters were related epithelial structure and function. Two clusters were upregulated from baseline to 6 mo. and included genes related to motile cilia and developmental signaling (Cluster 1: *TUBE1*, *CFAP47*, *LRP5*, *GSK3A*, *NUMB*) and ion homeostasis and transport (Cluster 3: *SCN4B*, *SLC6A13*, *SLC5A9*, *BEST4*). Conversely, the cluster related to squamous/cornified epithelium and keratinization (Cluster 4: *IVL*, *KRT13*, *SPRR3*) was downregulated from baseline to 6 mo. Finally, a cluster related cell cycle control and proliferation (Cluster 6: *TOP2A*, *CENPI*, *SPC24*, *ESCO2*) was mostly downregulated from baseline to 6 mo.

To identify functional gene networks based on shared expression throughout the time course, weighted gene coexpression network analysis (WGCNA) (27) was used to identify 12 coexpression modules (Table 1, Supplemental Figure 2, and Supplemental Table 5). The 5 inflammation- and immune function–related modules shared a pattern of decreased expression from baseline to 6 mo. The brown module was strongly enriched in neutrophilic inflammation–related genes (*CXCL8*, *TLR4*, *S100A8*, *IL1R2*), while the tan module contained genes belonging to the Toll-like receptor (TLR), IL-1, and TGF-β signaling pathways (*THBS1*, *MEF2C*, *MAPK14*, *IL6R*). The blue, red, and black modules corresponded to functions related to innate immunity (*MYD88*, *LCN2*, *IRAK2*, *APOE*), humoral adaptive immunity (*CD79A*, *LAMP3*, *IGHA1*, *IGKC*), and cell-mediated immunity (*CD4*, *CD8A*, *IL21R*, *IL32*), respectively. Three modules described the epithelial remodeling response with increased expression from baseline to 6 mo. The turquoise and magenta modules corresponded to motile ciliogenesis (*FOXJ1*, *RFX3*, *IFT88*, *DNAH5*) and ciliary signaling (*PKD1*, *WDR90*, *AHI1*, *CCDC57*), respectively. The yellow epithelial signaling module contained genes related to the Wnt, Hippo, epidermal growth factor, and other developmental signaling pathways (*WNT3A*, *WNT2B*, *YAP1*, *EGFR*) key to epithelial differentiation. The remaining modules displayed mixed patterns of expression and corresponded to lipid biosynthesis, lysosomal function, and ion homeostasis (green: *SMPD1*, *FABP5*, *LAMP1*, *SCNN1A*), mRNA splicing (pink: *SFSWAP*, *SRRM2*, *PRPF38B*, *RSRP1*), the cytoskeleton (green-yellow: *TUBG1*, *CKAP2*, *BFSP1*, *CILK1*), and organelle function (purple: *RPL5*, *RPS15*, *EIF3E*, *NOP53*). In summary, these data indicate that initiating ETI therapy results in wide-ranging cellular programming changes in both the epithelial and immune compartments that reflect substantially decreased inflammation accompanied by improved airway epithelial structure and mucociliary function for the baseline to 6 mo. timespan.

### Sinonasal transcriptional response from 6 mo. to 2 yr. reflects increased neutrophilic inflammation.

Interestingly, the 6 mo. to 2 yr. gene expression changes revealed 595 upregulated genes (adj. *P* < 0.05), which indicated a modest, but significant, upregulation of inflammatory pathways, including genes that were downregulated from baseline to 6 mo. (Figure 1E and Supplemental Table 3). This resurgence was also illustrated at the single gene expression level (*CXCR4*, *SERPINA1*, *TGFB1*, *TNF*, and others; Figure 2B). It was further corroborated by hierarchical gene clustering analysis where Clusters 2, 5, and 7 comprised of genes related to neutrophil degranulation and cytokine signaling, T cell activation, and inflammatory signaling, respectively, increased from 6 mo. to 2 yr. (Figure 2A). Moreover, the red, black, and tan WGCNA modules related to humoral adaptive immunity, cell-mediated immunity, and inflammatory signaling pathways, respectively, also displayed increased expression from 6 mo. to 2 yr. (Table 1). Finally, increased inflammatory gene expression was also observed at the cohort level by PCA, which showed that the displacement along the first principal component observed at 6 mo. compared with baseline was partially reversed at 2 yr., where the first principal component was defined by inflammation- and immune cell–related genes (*IL1B*, *CXCL8*, *FCGR3B*, *CXCR1*, *CXCR2*; Supplemental Figure 3, A and B, and Supplemental Table 6).

Interestingly, despite increased inflammatory gene expression, epithelial gene expression, which increased from baseline to 6 mo. to reflect improved epithelial structure and function, did not decrease from 6 mo. to 2 yr. Hierarchical clustering indicated no substantial changes in gene expression in Clusters 1, 6, and 4 related to motile cilia and ciliated cells, cell cycle and proliferation, or squamous epithelium, respectively (Figure 2A and Supplemental Table 4). This was consistent with no changes in the motile ciliogenesis and ciliary signaling (turquoise and magenta) or the epithelial signaling (yellow) WGCNA modules (Table 1, Supplemental Figure 2, and Supplemental Table 5). In sum, our data for the baseline to 6 mo. to 2 yr. timespan indicate that the initial, drastic decrease in inflammatory gene expression upon initiating ETI eventually began to resurge, although this resurgence was not accompanied by gene expression reflecting worsened epithelial structure and function. Of note, baseline sample collection spanned a 10-month period from September to July; thus, gene expression differences at 6 mo. or 2 yr. were unlikely to be driven by seasonal differences in exposures, rather they reflect the ongoing response to ETI.

### Changes in cellular composition are likely key drivers of the sinonasal airway response to ETI.

**Figure 3:**
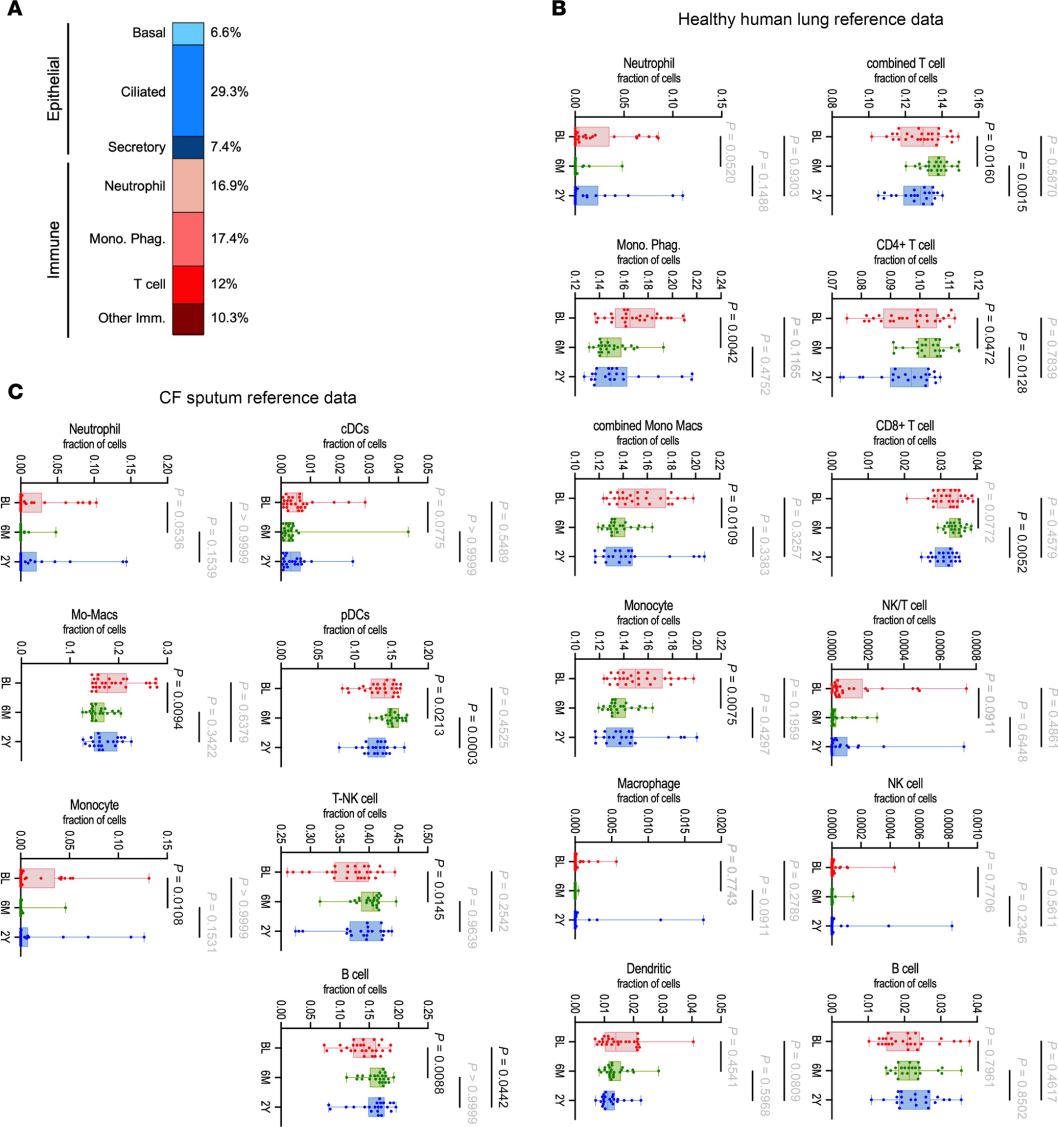
Cell type proportionality changes reflect a changing cellular landscape after ETI therapy. (**A**) Cell type enrichments of genes differentially expressed during the time course suggest major changes in mononuclear phagocytes, neutrophils, and ciliated cells. Computational deconvolution of immune cell type proportions based on healthy human lung (**B**) and CF sputum–derived (**C**) single-cell RNA sequencing reference datasets indicates an initial decrease in phagocytes and an increase in T cells upon ETI, which begins to reverse in the 6 mo. to 2 yr. span. Graphs show box-and-whisker plots with a Kruskal-Wallis test (**B** and **C**).

To understand what cell types may be driving the transcriptional response to ETI, we mapped the differentially expressed genes across the time course (Supplemental Table 2) to cell types and states identified in a healthy human lung single-cell RNA sequencing dataset (28). Nasal brush biopsies are mainly comprised of epithelial cells but also contain the immune cells that infiltrate or transit over the sampled epithelium. Correspondingly, differentially expressed genes mapped to epithelial cells including basal stem, secretory (mucous and non-mucous), and ciliated cell types and immune cells including neutrophils, mononuclear phagocytes (monocytes, macrophages, dendritic cells [DCs]), T cells, and other immune cell types (B cells, mast cells, eosinophils; Figure 3A). Consistent with the extensive transcriptional changes in immune and inflammatory processes, most (57%) of the mapped transcripts corresponded to immune cells, with the majority identified broadly as mononuclear phagocytes and neutrophils (both at 17%, Figure 3A). Furthermore, 29% of the differentially expressed transcripts across the time course mapped to ciliated cells, reflecting the extensive transcriptomic changes related to motile cilia upon ETI treatment (Figure 2A and Table 1).

Published healthy lung and CF sputum (Figure 3, B and C, and Supplemental Figure 4A) single-cell RNA sequencing reference datasets (28, 29) were used to computationally deconvolve the bulk RNA sequencing data to identify represented cell types and estimate cell type proportion changes over the time course. The 2 reference datasets contain partially overlapping immune cell types and produced similar results. The proportion of neutrophils trended toward decreasing and the proportion of monocyte/macrophage populations decreased significantly from baseline to 6 mo. and both trended toward increasing across the 6 mo. to 2 yr. time point. The opposite change was observed for T cells and plasmacytoid DCs (pDCs), which showed a significant increase from the baseline to 6 mo. and decrease across the 6 mo. to 2 yr. time points. Including the two 2 yr. transcriptional outliers in the analysis revealed that these samples were distinguished by an extremely high predicted proportion of neutrophils, monocytes, and macrophages, and a very low proportion of T cells, DCs, and epithelial cells consistent with the strong inflammatory signature in these samples (Figure 1B and Supplemental Figure 4B).

Epithelial cell changes showed a trend toward an increased proportion of ciliated cells from baseline to 6 mo. and no change between 6 mo. and 2 yr. and secretory cell types trended toward a decrease from baseline to 6 mo. and increase from 6 mo. to 2 yr. (Supplemental Figure 4A). The fraction of basal cells trended toward decreasing from 6 mo. to 2 yr., possibly reflecting an epithelium with reduced injury repair and turnover, which is consistent with the decrease in the proliferative gene signatures over the same timeframe (Figure 2A). Although future experiments are needed to validate the computational inference of cell type proportionality changes, these data suggest that there may be reduced recruitment of phagocytes and increased recruitment of T cells to the sinonasal airways in response to ETI, which begins to reverse despite ongoing therapy.

### Sinonasal epithelial transcriptomic responses correlate with airway disease clinical outcomes following ETI treatment.

**Figure 4:**
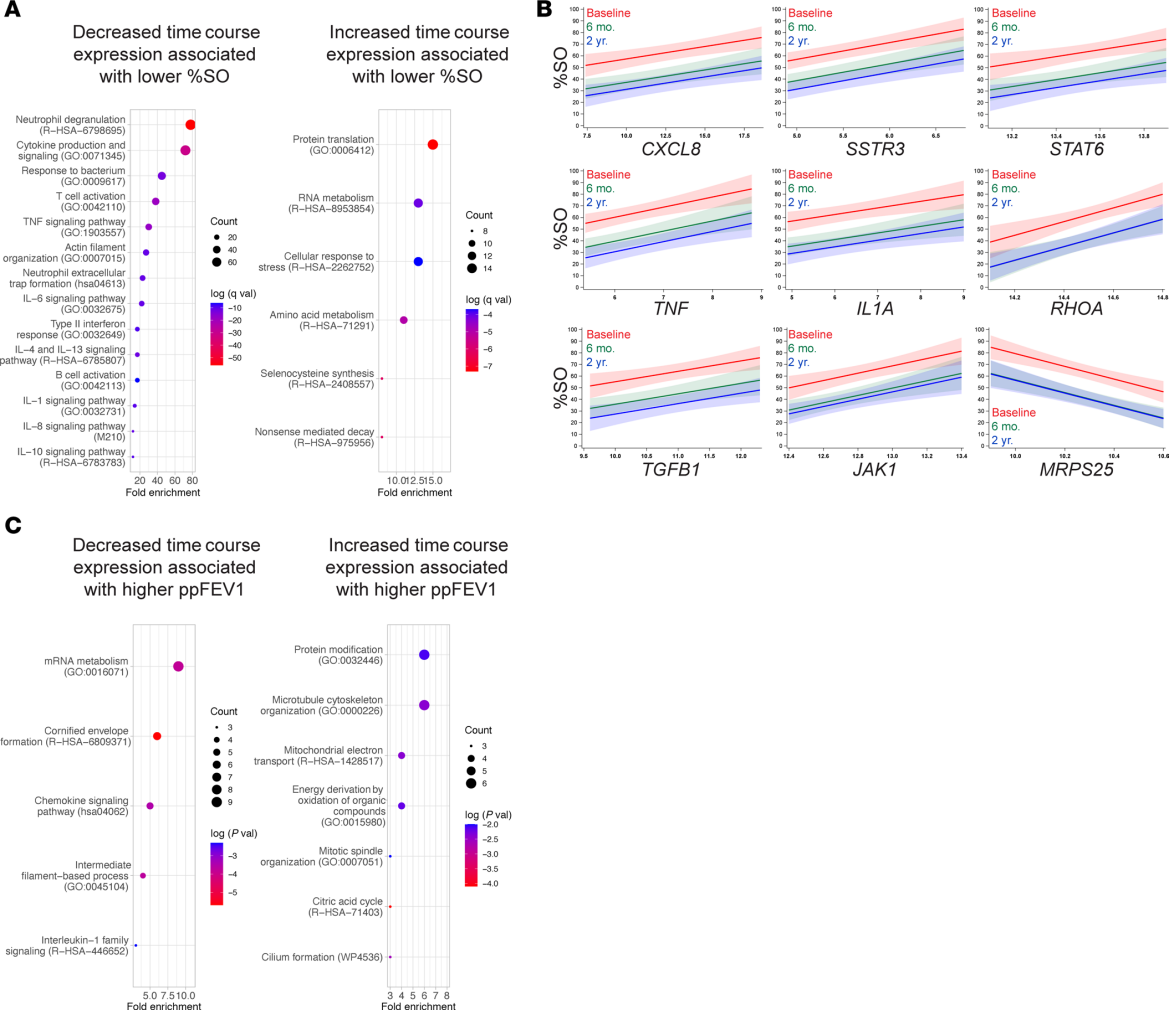
Time course gene expression is associated with clinical outcomes related to sinus disease and respiratory function after ETI therapy. (**A**) Pathway enrichments for genes whose decreased (left) or increased (right) expression is associated with lower %SO. (**B**) Association plots for select single genes of interest and %SO across the time course. The 6 mo. and 2 yr. data are nearly overlapping for *RHOA* and *MPRS25*. Each panel shows for a particular gene the predicted %SO (95% CI) as a function of gene expression (VST) derived from the linear mixed effects models that adjusted for time (excluding the time by gene expression interaction). (**C**) Pathway enrichments for genes whose decreased (left) or increased (right) expression across the time course is associated with higher ppFEV1.

Previously published clinical outcomes for this cohort of pwCF demonstrated substantial improvements in CF-related CRS and in lung function from baseline to 6 mo., with no significant cohort level changes from 6 mo. to 2 yr. (16–23). The percentage of the opacified 3-dimensional sinus volume calculated from computed tomography (%SO) (30) decreased an average of 21.7 points from baseline to 6 mo. (62.5 ± 4.0 to 40.8 ± 3.1, *P* < 0.0001) and was not significantly different from 6 mo. to 2 yr. (40.8 ± 3.1 to 36.6 ± 3.2, *P* = 0.82; Supplemental Figure 5A). Association analysis adjusted for time between %SO levels and differentially expressed genes across the time course (Supplemental Table 2) showed that lower %SO, an indication of reduced CRS severity, was significantly (adj. *P* < 0.05) associated with the decreased expression of 470 genes. These were enriched in pathways related to neutrophil activation (*FCGR2A*, *CXCR1*, *CXCR2*, *SERPINA1*), neutrophil extracellular trap formation (*AQP9*, *C5AR1*, *ITGAM*, *PADI4*), and cytokine production and signaling (*IL1A*, *CXCL8*, *TNF*, *TGFB1*; Figure 4, A and B, and Supplemental Table 7). The top 25 genes whose reduced expression was most strongly associated with lower %SO included *JAK1* (Janus kinase 1), *STAT6* (signal transducer and activator of transcription 6), and *GNS* (glucosamine N-acetyl-6-sulfatase), which may be related to downregulation of airway epithelial mucus hypersecretion, a key target in CF disease attenuation by ETI therapy. Lower %SO was also significantly (adj. *P* < 0.05) associated with increased expression of 100 genes that were enriched in pathways related to RNA processing and protein translation (*MRPS25*, *TSEN34*, *PCBP1*, *RPS11*) and mitochondrial function and oxidative stress response (*TOMM7*, *LDHD*, *SUCLG2*, *NDUFAF2*; Figure 4A). Increased expression of these genes may reflect normalization of epithelial structure and function in response to ETI. Association analysis between gene expression and %SO at distinct time points (baseline, 6 mo., or 2 yr.) or the change in %SO between specific time points (baseline to 6 mo. or 2 yr., or 6 mo. to 2 yr.) produced strongly overlapping outcomes with the above at *P* < 0.05 (Supplemental Figure 5, B and C, and Supplemental Table 7).

ppFEV1, the chief metric of lung function in pwCF, rose an average of 11.9 points from baseline to 6 mo. (65.6 ± 4.7 to 77.5 ± 5.7, *P* = 0.01) and was not significantly different from 6 mo. to 2 yr. (17) (77.5 ± 5.7 to 79.8 ± 5.2, *P* = 0.36; Supplemental Figure 5A). ppFEV1 association analysis yielded only a few genes that passed the significance threshold of an adjusted *P* value of less than 0.05. However, due to the modest sample size and the complexity of the ppFEV1 metric, genes with an unadjusted *P* value of less than 0.05 may still represent biologically meaningful candidates. Reduced expression of 93 genes was associated with higher ppFEV1, an indication of better lung function. These were enriched in pathways related to squamous/cornified epithelium (*KRT13*, *DSC3*, *DSG3*, *EVPL*) and IL-1 family signaling (*IL1RL1*, *CSF1R*, *RELA*, *PRKACA*; Figure 4C and Supplemental Table 8). The 100 genes whose increased expression was associated with higher ppFEV1 included those related to motile ciliogenesis (*CEP44*, *IFT20*, *KIZ*, *TEKTIP1*) and mitochondrial oxidative phosphorylation (*SUCLG2*, *ISCA1*, *SDHAF4*, *LYRM2*; Figure 4C and Supplemental Table 8). These data suggest that increased lung function may be associated with a compositionally more normal epithelium with more ciliated and fewer squamous/cornified cells and lower IL-1–dependent inflammation.

### Baseline sinonasal epithelial gene expression may predict clinical response to long-term ETI therapy.

**Figure 5:**
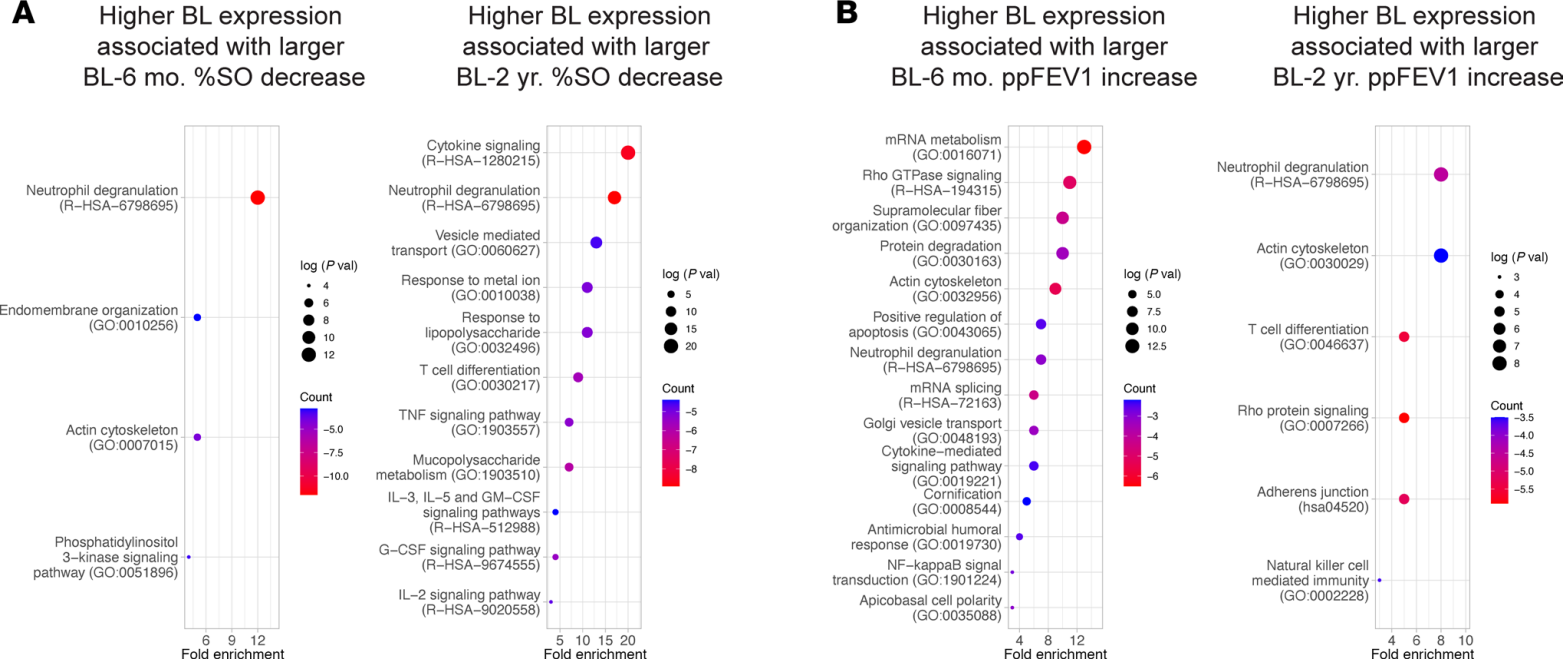
Sinonasal airway epithelial gene expression at baseline is associated with clinical outcomes related to sinus disease and respiratory function after ETI therapy. (**A**) Pathway enrichments for genes whose higher baseline expression is associated with a larger decrease in %SO from baseline to 6 mo. (left) and 2 yr. (right). (**B**) Pathway enrichments for genes whose higher baseline expression is associated with a larger increase in ppFEV1 from baseline to 6 mo. (left) and 2 yr. (right).

Association between baseline gene expression and 6 mo. or 2 yr. %SO and ppFEV1 was tested with the caveat that about half of the participants (16 of 30, Supplemental Table 1) had received less effective CFTR modulators prior to ETI. A greater baseline to 6 mo. decrease in %SO was significantly associated (*P* < 0.05) with higher baseline expression of genes related to innate immune response and inflammatory signaling (*OSM*, *PTX3*, *S100A12*, *PGLYRP1*, *GPR84*) and actin cytoskeleton and intracellular trafficking (*RHOA*, *VASP*, *RAB5C*, *IQGAP1*; Figure 5A and Supplemental Table 9). A similar set of genes associated with a greater baseline to 2 yr. decrease in %SO included those related to innate immune response (*ORM1*, *PTX3*, *PGLYRP1*), immune cell recruitment and trafficking (*GPR84*, *CXCR4*, *ICAM1*, *PECAM1*), cytokine signaling (*JAK1*, *STAT5A*, *NFKB2*, *TGFB1*, *IL1A*), oxidative stress and mitochondrial function (*SOD2*, *MICU1*, *NADK2*, *GPD2*), and actin cytoskeleton (*RHOA*, *CD14*, *FGF11*, *NRAS*; Figure 5A and Supplemental Table 9). Meanwhile, lower baseline expression of genes related to protein translation (*EIF3G*, *ANKZF1*, *SAYSD1*, *MRPS25*) was correlated with a greater decrease in %SO from baseline to 6 mo. and protein translation (*EEF1D*, *EIF2D*, *RPL4*, *RPL5*), and Notch signaling (*YBX1*, *MIB2*, *SPTAN1*, *ACIN1*, *ITGB1BP1*) from baseline to 2 yr. (Supplemental Figure 6A and Supplemental Table 9). WGCNA modules (Table 1) also identified a correlation (*P* < 0.05) between absolute %SO values at 6 mo. or 2 yr. or changes in %SO from baseline to 6 mo. or 2 yr. Significant correlations were identified for the red (humoral adaptive immunity), tan (inflammatory signaling), brown (neutrophilic inflammation), and purple (organelle function) modules (Table 2). In addition to multiple inflammation-related genes, both analyses revealed a correlation between genes in the Notch signaling pathway, a central regulator of airway epithelial cell fate and %SO outcomes (gene based approach: *YBX1*, *RPS27A*, *MIB2*, *SPTAN1*, *ACIN1*, *ITGB1BP1*, *EPN2*, *TSPAN15*; WGCNA based approach: *NLE1*, *POGLUT1*, *OTUD5*, *PSMD2*, *TLE4*, *HDAC1*, *ATXN1*). While additional validation and mechanistic studies are needed, these data suggest that baseline Notch activity may be correlated with CRS outcomes after ETI.

Association analysis revealed that a greater baseline to 6 mo. increase in ppFEV1 was significantly associated (*P* < 0.05) with higher baseline expression of genes related to immune response and inflammation (*MIF*, *IL1RL1*, *CSF1R*, *RELA*, *S100A2*, *S100A10*) and actin cytoskeleton regulation (*RHOA*, *CFL1*, *SPTAN1*, *CAPG*, *FLII*; Figure 5B and Supplemental Table 10). A highly overlapping set of genes was associated with a greater baseline to 2 yr. increase in ppFEV1 and included those related to immune response and inflammation (*CXCR5*, *IL1RL1*, *NFKBID*, *S100A10*), actin cytoskeleton regulation (*RHOA*, *CFL1*, *ARHGAP4*, *CAPZB*), and squamous/cornified epithelium (*KRT6A*, *IVL*; Figure 5B and Supplemental Table 10). In both cases, a greater increase in ppFEV1 was associated with higher baseline *POU2F3*, a transcription factor of the rare cell lineage that includes CFTR-rich ionocytes (31). Genes whose lower baseline expression was associated with a greater increase in ppFEV1 included those related to lipid metabolism (*SCP2*, *INSIG2*, *CEL*) and mitochondrial function (*PPA2*, *PMPCB*, *ALDH6A1*, *ALDH5A1*, *COQ3*) for baseline to 6 mo., and those related to lipid metabolism (*INSIG2*, *SLC2A13*, *CRBN*), mitochondrial function (*PPA2*, *COQ3*, *UQCRFS1P1*, *NFS1*), and proliferation (*ACTL6A*, *CENPE*, *CENPF*, *GEN1*) for baseline to 2 yr. (Supplemental Figure 6B and Supplemental Table 10). WGCNA-based analysis identified a significant (*P* < 0.05) correlation for the black (cell-mediated immunity) and green (cellular metabolism) modules (Table 3). These data suggest that baseline expression that reflects increased inflammation may be associated with increased ppFEV1 after initiating ETI.

### Host transcriptomic responses to ETI are accompanied by changes in the sinonasal microbiome.

**Figure 6:**
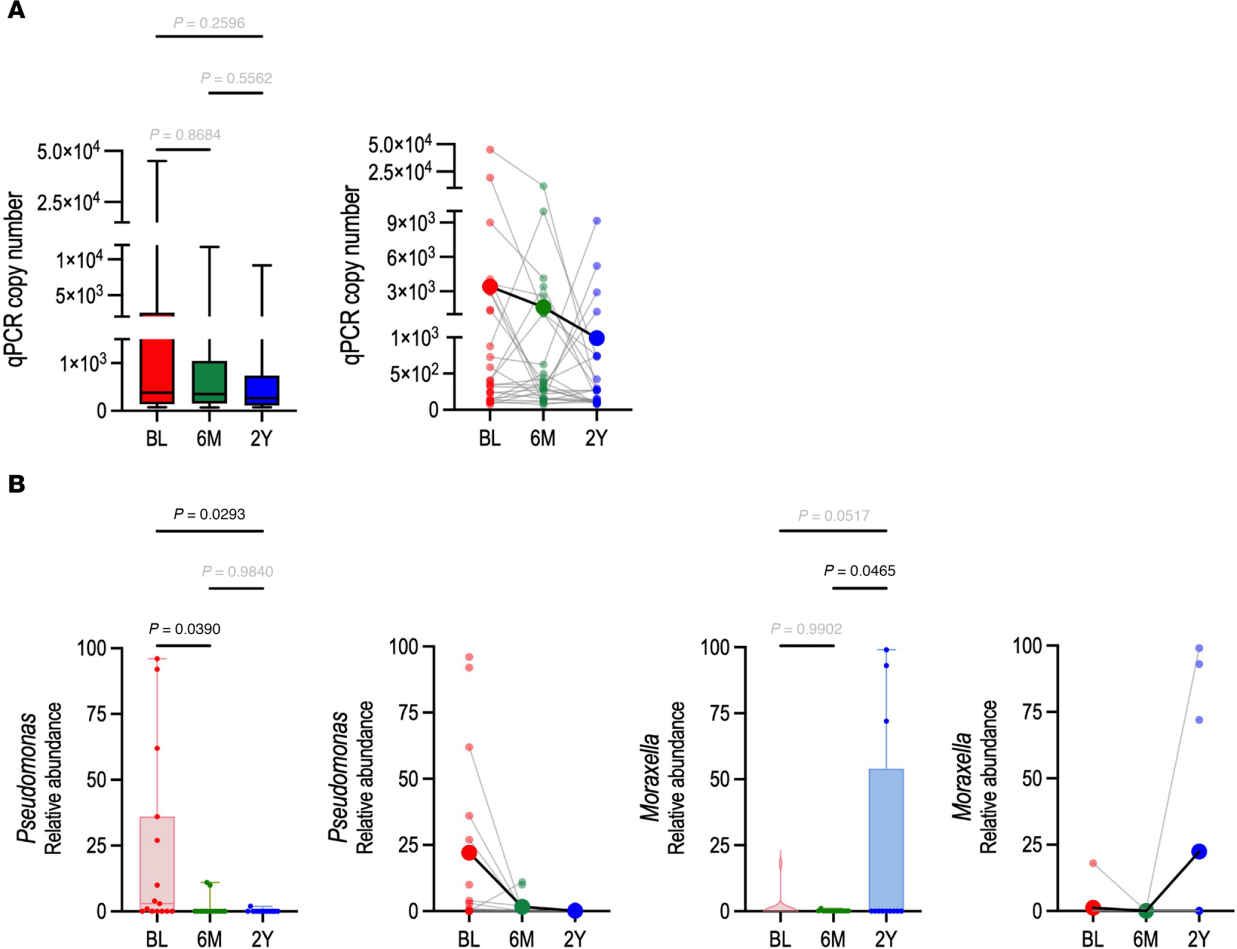
Sinonasal microbiome changes in response to ETI therapy. (**A**) Microbial abundance indicated by 16S rRNA copy number trends toward decreasing during the time course. Data are shown excluding 2 yr. outliers. Left: Box-and-whisker plot with 1-way ANOVA followed by Tukey’s post hoc test. Right: Individual data points throughout time course overlaid with per–time point mean. (**B**) Relative abundance among the top 25 sequenced taxa indicates a significant decrease in *Pseudomonas* (left) and an increase in *Moraxella* (right) during the time course. Significance assessed with 1-way ANOVA followed by Tukey’s post hoc test.

Substantial airway microbiome changes have been documented in response to ETI therapy, including a drastic decrease in, but not eradication of, common CF airway pathogens (6–13). To assess changes upon ETI initiation, we characterized viral and microbial species in the nasal brush biopsies. A computational approach to identify viral sequences in whole transcriptome RNA sequencing data detected viral reads above threshold in only a few samples. Rhinovirus C was detected in baseline samples from 3 participants (Supplemental Table 11). None of the 6 mo. or 2 yr. samples were positive for any respiratory virus. The microbiome of the inferior turbinate was assessed by 16S ribosomal RNA amplification and sequencing using genomic DNA copurified with the RNA from the brush biopsies. Amplification was successful for all samples. Mean qPCR copy number as a metric of overall microbial abundance decreased substantially over the time course, although it did not reach statistical significance (Figure 6A).

Due to the limited amount of material obtained from the nasal brushes, taxon-level sequencing was successful for only 15 baseline, thirteen 6 mo., and thirteen 2 yr. samples comprising 7 complete time courses, 11 baseline–6 mo. pairs, seven 6 mo.–2 yr. pairs, and 7 baseline–2 yr. pairs. *Staphylococcus*, *Moraxella*, *Pseudomonas*, *Corynebacterium*, and *Propionibacterium* were the most abundant within the top 25 detected taxa (Supplemental Table 12). Detected taxa included both those that contain species that are considered to be a part of the nasal commensal microbiota (*Corynebacterium*, *Dolosigranulum*, *Anaerococcus*) and those that contain CF-associated species (*Staphylococcus*, *Pseudomonas*, *Streptococcus*, *Prevotella*). Importantly, the relative abundance of *Pseudomonas* decreased significantly over the time course (Figure 6B). This may indicate a marked reduction in *Pseudomonas aeruginosa*, the dominant CF airway pathogen in adults in response to ETI, which has been documented by other studies (6–13). Interestingly, the relative abundance of *Moraxella* was very high in some 2 yr. samples (Figure 6B). Changes in the relative abundance of other taxa or in microbial diversity over the time course were not significant (Supplemental Figure 7, A and B). Importantly, we failed to detect increased viral or bacterial pathogens at 2 yr., suggesting that a rebound in pathogens after 6 mo. of ETI therapy is likely not the main driver of the resurgence of host inflammatory gene expression described above (Figure 1E). The two 2 yr. transcriptional outlier samples contained no detectable viral species, although one showed a high abundance of bacterial taxa but not a higher relative abundance of *Pseudomonas* (Supplemental Figure 7, C and D). Thus, we cannot confirm that an ongoing infection is the cause of the outlier status. These samples may represent a stronger return to baseline inflammation that would require a richer sample set to fully characterize.

### Primary sinonasal airway epithelial cell cultures derived from donors after ETI therapy have mostly improved structure and function.

**Figure 7:**
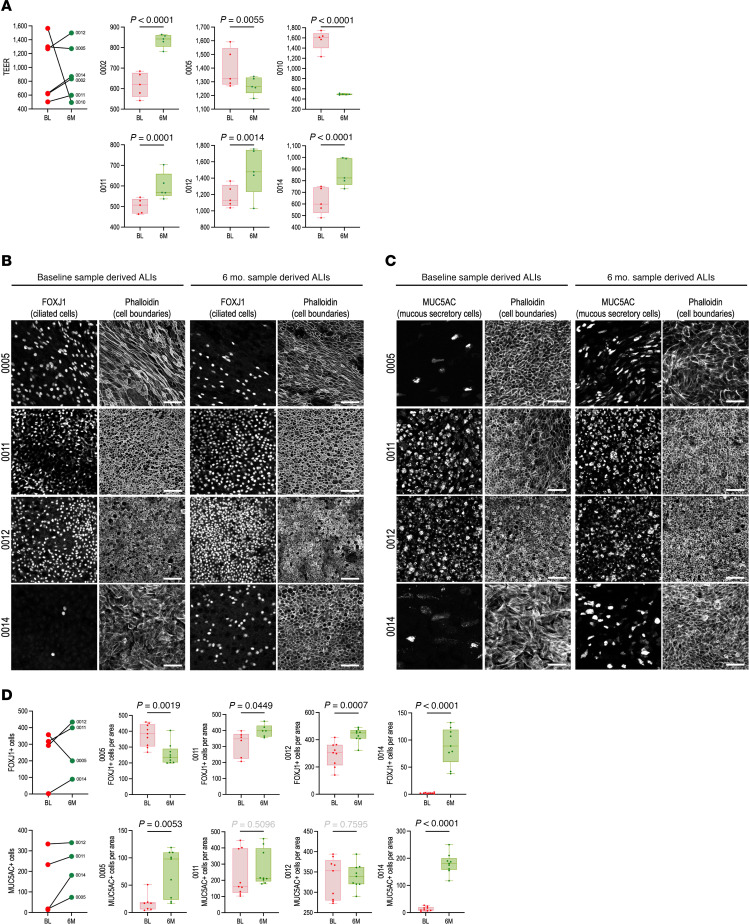
Improved epithelial structure and function in primary sinonasal airway epithelial cultures after ETI therapy. (**A**) Matched baseline and 6 mo. primary sinonasal airway epithelial air-liquid interface (ALI) cultures indicate increased barrier capacity at 6 mo. in most participants as measured by transepithelial electrical resistance (TEER, Ω·cm^2^). Individual participant graphs show the average of 3 measurements from *n* = 5 independent cultures. Graphs show box-and-whisker plot with 2-tailed *t* test. (**B**) ALIs from matched baseline and 6 mo. samples stained with phalloidin for cell boundaries and labeled with anti-FOXJ1 antibody to mark ciliated cells show improved differentiation at 6 mo. in ALIs with increased TEER. Scale bars: 50 μm. (**C**) ALIs from matched baseline and 6 mo. samples stained with phalloidin for cell boundaries and labeled with anti-MUC5AC antibody to mark mucous secretory cells show improved differentiation at 6 mo. in some ALIs. Scale bars: 50 μm. (**D**) Quantitation of FOXJ1- and MUC5AC-positive cells. Individual graphs show the average of at least 3 measurements from *n* = 3 independent cultures. Graphs show box-and-whisker plots with 2-tailed *t* test.

CF basal stem cells are characterized by an “inflammatory memory” such that basal cell populations isolated from the CF airways retain functional defects and compositional changes in in vitro culture (32–35). This stem cell dysfunction is considered to be an important manifestation and a driver of epithelial remodeling. To determine whether the transcriptional changes described above in fact manifest over time in a less remodeled epithelium after initiating ETI therapy, basal stem cells were isolated from nasal brush biopsies at baseline and 6 mo. after ETI (Figure 1A) and differentiated in primary culture. Due to COVID-19 pandemic–related research restrictions that went into effect shortly after starting the 6 mo. sample collection, matched pairs of baseline and 6 mo. samples were only available from a limited number of participants to generate primary air-liquid interface airway epithelial cell cultures (ALIs). No significant differences were observed between the baseline and 6 mo. samples in the total cell counts per brush (1.46 × 10^6^ ± 0.22 × 10^6^ at baseline vs. 1.51 × 10^6^ ± 0.18 × 10^6^ at 6 mo., *P* = 0.82), the total number of airway epithelial basal cells after expansion (3.81 × 10^6^ ± 0.65 × 10^6^ at baseline vs. 3.49 × 10^6^ ± 0.17 × 10^6^ at 6 mo., *P* = 0.65), or the number of days required for basal cell expansion in culture (6.17 at baseline vs. 6.55 at 6 mo., *P* = 0.40). To model in vitro CFTR modulation, both baseline and 6 mo. ALIs were also treated with commercially obtained ETI compounds. Epithelial barrier capacity of mature ALIs measured by transepithelial electrical resistance (TEER) was significantly improved at 6 mo. compared with baseline in 4 out of the 6 cultures (Figure 7A), although measurements remained substantially lower than for ALIs derived from healthy control donors (Supplemental Figure 8A). ETI treatment of ALIs during differentiation had a variable effect on TEER (Supplemental Figure 8B), and thus ETI is likely not sufficient on its own or during such a short timeframe to improve epithelial function. Changes in the extent of mucociliary differentiation were assessed by quantitating ciliated and MUC5AC^+^ mucous secretory cell numbers. Ciliated cell numbers mirrored the changes in TEER such that ALIs with increased TEER at 6 mo. also contained more ciliated cells (Figure 7, B and D). Mucous secretory cell numbers were either increased or not significantly changed in the 6 mo. cultures compared to baseline (Figure 7, C and D). Overall, these data suggest that the sinonasal basal cell pool from some pwCF returns to a healthier phenotype after ETI with improved mucociliary differentiation, while others retain the dysfunction induced by the CF environment.

## Discussion

Consistent with substantial improvements in clinical and patient reported outcomes and decreased detection of *Pseudomonas*, transcriptomic changes in the sinonasal airway mucosa reflect massively decreased neutrophilic inflammation and improved epithelial remodeling at 6 mo. after initiating ETI therapy in an adult cohort of pwCF. These integrated responses mirror studies reporting rapid and stable improvements in lung and sinus disease accompanied by decreased inflammation and infection. Transcriptomic changes were dominated by inflammation- and immune cell–related genes and processes. These included virtually all key players and pathways known to drive CF airway inflammation in response to infection and epithelial injury downstream of defective CFTR-dependent ion transport and mucociliary dysfunction (2), indicating that this dataset represents a comprehensive atlas of the global molecular changes that take place in the airway mucosa upon restoring CFTR function. Consistent with neutrophil dominant inflammation in CF, the most significant gene expression changes upon ETI reflected a major decrease in neutrophil-specific gene expression, including markers of neutrophil activation (*TLR4*, *CXCR1*, *CXCR2*) (36). This finding, together with decreased expression of neutrophil chemoattractants (*CXCL8*, *CXCL1*, *CXCL2*) suggests that fewer neutrophils are recruited to the sinonasal mucosa, which agrees with studies demonstrating fewer neutrophils and lower neutrophil elastase in sputum after ETI (37). We also observed reduced expression of genes related to neutrophil extracellular trap formation (*PADI4*, *NCF4*), which was also demonstrated in the lower airways in response to ivacaftor monotherapy (38), a very effective CFTR modulator therapy for pwCF carrying the G551D or related variants. We also found evidence for decreased activation and abundance of macrophages based on lower expression of *TNF*, *IL1R2*, *CCL3/4*, *NLRP3*, and *AQP9* (encoding aquaporin 9), a regulator of IL-1β secretion, which was previously shown to be decreased by ETI in pediatric study participants (39). Accompanying the loss of neutrophils and macrophages, the dataset provides evidence for increased infiltration of T cells upon ETI therapy, which may be related to the increased regulatory T cells in peripheral blood documented in response to ETI (40).

A similar study probing the sinonasal airway response to ETI uncovered only a handful of significant transcriptomic changes, although these did include decreased expression of inflammation- and mucin secretion–related genes (*FCGR1A*, *TGFBI*, *GAL3ST4*) (14). The modest number of changes in that study may have been due to comparing baseline expression to a wide range of time points spanning 3–12 months after ETI in a small, mixed adult and pediatric study population. Regardless, both studies support the conservation of key pathways and players between sinonasal and lower airway disease in CF. Independently, there also is a need for deeper understanding of sinonasal biology in CF, as sinus disease remains a major concern for pwCF (41) and the sinonasal epithelium remains understudied.

Decreased inflammation upon restoring CFTR function is thought to occur due to the drastic reduction in airway pathogens upon improved mucociliary clearance (42). Data showing decreased bacterial abundance and specifically decreased *Pseudomonas* relative abundance during the time course are consistent with larger analyses that detected a decrease in CF-related pathogens and shift toward normalization of the nasal and lower airway microbiome with ETI therapy (6–13). Importantly, studies highlight that eradication of *Pseudomonas* and other pathogens is not achieved.

The epithelial cellular response to ETI showed a substantial increase in motile cilia– and ciliated cell–related transcripts and a decrease in those related to mucous and squamous metaplasia consistent with reduced remodeling. The loss and dysfunction of ciliated cells is well documented although underappreciated in CF (33, 43, 44) as a driver of mucociliary defects in addition to the buildup of thick, pathogenic mucus. Increased expression of ciliary genes and imputed proportions of ciliated cells and the strong inverse correlation between ciliary and inflammatory gene expression suggest that more functional ciliated cells are restored to the CF airways as inflammation subsides. Downregulation of proinflammatory mediators, such as IL-1β that drives mucous metaplasia (45), may be critical for the normalization of cellular differentiation pathways to produce a more optimal balance of ciliated and secretory cells. Extensive changes in the Wnt, Notch, and Hedgehog signaling pathways that control epithelial differentiation likely reflect fundamental shifts in injury repair programs to reduce remodeling.

To directly address whether ETI improves epithelial basal stem cell defects (32–35), the in vitro differentiation capacity of basal cells isolated at baseline and 6 mo. from matched donors was compared. ALIs revealed that most, but not all, 6 mo. donor basal cells produced a structurally and functionally more normal epithelium. ALIs from 6 mo. still contained fewer ciliated cells and had reduced barrier function compared with healthy donor ALIs, indicating that the inflammatory memory that characterizes basal cell dysfunction is retained. This is consistent with this and multiple other studies observing that inflammation is not fully rectified by ETI (37, 46), which has potentially major implications for long-term therapy. Addition of ETI compounds to ALIs failed to improve differentiation. A similar study using transcriptomic analysis also only identified a few differentially expressed genes accompanying increased CFTR-dependent chloride transport, which were related to bacterial defense (*DEFB1*) and cellular stress response (*HMOX1*) (15). This finding suggests that normalization of the airway stem cell pool may require other interventions, longer timeframes, or input from cells not included in the ALI model.

Transcriptomic data for the 6 mo. to 2 yr. timeframe indicated an increase in inflammation- and immune cell–related gene expression, consistent with a modest resurgence of the neutrophilic inflammation that decreased from baseline to 6 mo. Despite no significant change in CRS or pulmonary function, this finding may represent early evidence of disease progression after ETI therapy. This observation is consistent with long-term outcomes after ivacaftor monotherapy, for which rapid and substantial improvements were followed by eventual disease progression (47, 48). In these participants, bacterial abundance increased first followed by a rebounded inflammatory response (49). In this cohort, bacterial or viral pathogens were not increased between 6 mo. and 2 yr. Since levels of inflammatory mediators were not possible to evaluate at the protein level, it remains unclear whether the increased inflammatory gene expression translates to a rebounded inflammatory response that may be of clinical significance.

A highly significant association was established between sinonasal gene expression and the extent of CF-related CRS quantified as %SO by computed tomography. Genes whose reduced expression associated with lower %SO, indicating less severe disease, included genes that are known biomarkers of CF airway disease such as IL-8 (*CXCL8*) and TNF-α (*TNF*) and candidate biomarkers such as *CD14*, *CEACAM3*, *TGFB1*, and *TLR4* previously identified in the lower airways (50–52). These data delineate clinically and biologically significant molecular players and programs, and provide specific candidates for mechanistic studies. Genes associated with lower %SO included many cell-surface (*IGF2R*, *IL18R1*, *TNFRSF8*, *SSTR3*, *CD83*) and secreted (*IL1A*, *CXCL8*, *CXCL16*, *PROK2*, *OSM*) factors that will aid further validation and discovery at the protein level and in other tissues and specimens. Only nominally significant associations were detected between gene expression and ppFEV1; however, these include multiple biologically plausible targets. Higher ppFEV1, an indication of better lung function, was associated with decreased expression of inflammatory and squamous epithelial genes and increased expression of ciliary genes, reflecting a normalized airway mucosa. Reduced *HDAC1* association with higher ppFEV1 may be of interest, as HDAC inhibition has been shown to improve CFTR stability (53). No significant correlation between %SO and ppFEV1 was observed in the study cohort (20), likely reflecting the complexity of the ppFEV1 metric and the small sample size.

A previous study leveraging baseline sinonasal gene expression to predict clinical response to ETI proposed a transcriptomic risk score to serve as a predictive molecular biomarker for respiratory function and body mass index metrics (14). Of note, this study employed relaxed statistical parameters on a transcriptomic dataset that identified only a handful of significant gene expression changes between the baseline and ETI treatment time points. Our dataset largely failed to replicate these findings, identifying only a few overlapping genes (*CD244*, *CXCL10*, *DOK2*, *MYO1G*, *SLAMF6*) whose higher pre-ETI expression was associated with improvement in ppFEV1 after ETI. Only nominally significant associations were identified between baseline expression and 6 mo. or 2 yr. ppFEV1, although these chiefly belonged to inflammation-related biological pathways. Inflammatory mediators found in the CF airways such as TNF-α, IL-1β, and IL-17 have been shown to augment CFTR modulator efficacy in cultured cells (54, 55) and their higher baseline levels correlate with improved ppFEV1 after ivacaftor (54). This raises the possibility that increased baseline inflammation may also lead to an enhanced ETI response, which should be further investigated. Nonetheless, these datasets suggest that nasal brush biopsy transcriptomics should be further evaluated for the long-term surveillance of ETI response, and for the early detection and potential prediction of airway disease progression. Combined analysis of datasets may help establish robust predictive models of ETI response.

Several key limitations exist for this and similar studies on ETI response. First, the single-site study with a modest sample size may limit generalizability. Although only adult pwCF with established disease were studied, participants were heterogeneous in CFTR genotype and baseline lung and sinus disease severity. How the extent of baseline epithelial remodeling impacts ETI outcomes remains unclear and understudied. Furthermore, the overlap of the COVID-19 pandemic with the introduction of ETI is an important factor with unknown significance. Although SARS-CoV-2 was not detected in the study samples, the pandemic undoubtedly altered exposures not only to respiratory pathogens, but also potentially to pollutants and allergens. Finally, ETI initiation has been generally accompanied by extensive changes in or de-escalation of other treatments such as antibiotics (56), which may confound outcomes. Continued longitudinal studies and combinatorial analysis of datasets will be important to both validate and strengthen findings.

The sinonasal mucosal gene expression time course integrated with clinical outcomes and microbiome and host cellular changes in this study serve as a robust foundation for delineating the key molecular and cellular mechanisms that underlie and potentially predict treatment response to ETI. Application of similar longitudinal sampling to more effective CFTR modulators or future cell- and gene-based therapies will aid in better understanding of efficacy, surveillance of disease state, and prediction of disease progression.

## Methods

### Sex as a biological variable.

Both male and female participants were included in the study. Sex differences were not evaluated as part of the data analysis.

### Study participants, nasal brush biopsy, and nucleic acid and basal stem cell isolation.

Participants were previously described (16–23). Briefly, adults with CF who received ETI therapy for clinical purposes were recruited between August, 2019 and June, 2022 at National Jewish Health. Nasal brush biopsies were obtained serially from the same participant at baseline and at approximately 6 mo. and 2 yr. after initiating ETI therapy. Brushes from the right nostril were collected in Qiagen RLT Plus lysis buffer supplemented with 40 mM DTT and stored at –80°C. Total RNA and DNA were isolated simultaneously using the AllPrep DNA/RNA Mini Kit (Qiagen). The mean RNA integrity number (RIN) was 8.60 ± 0.07, which is indicative of highly intact RNA. Brushes from the left nostril were collected in airway epithelial cell culture medium (35). Cells were immediately seeded onto collagen I–coated plastic dishes for basal cell expansion. Basal cells were cultured in homemade proliferation medium supplemented with ROCK inhibitor (10 μM; Y-27632), BMP inhibitor (1 μM; DMH-1), TGF-β inhibitor (1 μM; A-83-01), and WNT agonist (1 μM; CHIR-99021) (all Selleckchem), and then cryopreserved at passage 1.

### Whole transcriptome sequencing and data analysis.

RNA sequencing libraries were trimmed with cutadapt v2.10 (https://github.com/marcelm/cutadapt) to remove adapters, short reads (–m 20), and low quality 3′ bases (–q 10) and quantified using salmon v1.4.0 (https://github.com/COMBINE-lab/salmon) with 50 bootstraps and a gencode v36 reference (https://www.gencodegenes.org). Salmon abundance estimates were imported into R v.4.3.2 (https://www.r-project.org) and converted to gene-level count estimates using tximport v1.30.0 (https://bioconductor.org/packages/release/bioc/html/tximport.html). Counts were transformed for visualization using the vst function from DESeq2 v1.42.0 (https://bioconductor.org/packages/release/bioc/html/DESeq2.html). Principal component analysis (PCA) was performed in R using the 500 most variable genes and visualized with the ggplot2 v3.5.1 package (https://cran.r-project.org/web/packages/ggplot2/index.html) . PCA revealed a strong sex-specific effect, which was effectively removed using Combat-seq (part of the sva package, v3.50.0; https://bioconductor.org/packages/release/bioc/html/sva.html). Technical replicates across experimental batches confirmed the lack of a significant experimental batch effect, so technical replicates were averaged. Differential expression testing across experimental time points was performed on raw counts using a negative binomial mixed model with adaptive Gaussian quadrature parameter estimation and bootstrapping (1000 samples) on read counts to empirically calculate *P* values as described previously (57). This approach used the GLMMAdaptive package v0.8-8 (https://cran.r-project.org/web/packages/GLMMadaptive/index.html). Modeling was performed treating time as either a continuous variable (LRT test), a continuous variable with a natural spline fit with 2 degrees of freedom (LRT), or a categorical variable (pairwise Wald’s test). Multiple testing correction was accomplished using the Benjamini-Hochberg false discovery rate (FDR) approach. WGCNA v1.72-5 (https://cran.r-project.org/web/packages/WGCNA/index.html) was carried out to identify co-regulated genes across the dataset. Cell type deconvolution was performed using the InstaPrism v0.1.5 package (https://github.com/humengying0907/InstaPrism) with published single-cell RNA sequencing datasets as reference (28, 29). Cell proportion estimates were visualized using ggplot2.

### Association analysis with clinical outcomes.

Collection and analysis of clinical data for the study participants were previously described (16–23). Briefly, sinus opacification as a metric of sinus disease was assessed via a deep learning algorithm from computed tomography (30). %SO represents the percentage of the sinus cavities filled with fluid and thickened mucosa, with higher values corresponding to worse disease. ppFEV1 as a metric of lung function was calculated from spirometry, with lower values corresponding to worse disease. Genes associated with %SO were identified by first calculating Pearson’s correlation coefficients between the clinical variable and all WGCNA module Eigengenes. LASSO regression was then performed with the genes from each WGCNA module with significant correlation (*P* ≤ 0.05) using the glmnet package v4.1-8 (https://cran.r-project.org/web/packages/glmnet/index.html) to identify genes predictive of clinical outcomes. For genes that were differentially expressed over time, the association between clinical outcomes of interest (%SO and ppFEV1) and gene expression was examined using linear mixed effects models (LMMs). To examine the overall relationship between clinical outcomes of interest and gene expression while accounting for changes in each outcome over time, we constructed LMMs with terms for study visit (baseline, 6 mo., and 2 yr.) and gene expression. To examine the relationship between gene expression and the change over time in clinical outcomes of interest, we constructed LMMs with terms for study visit, gene expression, and a visit by gene expression interaction term. All LMMs included a random subject intercept to account for correlated observations. Raw read counts for gene expression were transformed using a variance stabilizing transformation (VST) to account for differences in sample library sizes and to scale gene expression to the outcomes of interest. Benjamini-Hochberg FDR-adjusted *P* values were used to assess statistical significance, which was considered at the α = 0.05 significance level. Gene expression transformations were conducting using DESeq2 in R, and all LMMs were fit in SAS 9.4 (SAS Institute). Single gene association plots depict estimated mean (95% CI) %SO as a function of gene expression (VST scale) adjusting for time. Plots were generated by fitting a mixed model on %SO with a term for gene expression (VST) and time (categorical) then using that model to estimate the expected %SO (and 95% CI) along the range of gene expression (on the VST scale) values for each time point.

### Microbiome analysis.

Raw RNA sequencing data were analyzed with VirProf (http://github.com/seiboldlab/virprof) software version 0.8.1 for recovering and quantifying virus genomes in transcriptomic data. Default settings were applied, depleting host reads using the Gencode human genome (hg38) version 38. Bacterial profiles were determined by broad-range amplification and sequence analysis of 16S rRNA genes following previously described methods (58, 59). Briefly, amplicons were generated using primers that target approximately 300 base pairs of the V1V2 variable region of the 16S rRNA gene. PCR products were normalized using agarose gel densitometry, pooled, lyophilized, purified, and concentrated using a DNA Clean and Concentrator Kit (Zymo). Pooled amplicons were quantified using a Qubit Fluorometer 2.0 (Invitrogen). The pool was diluted to 4 nM and denatured with 0.2N NaOH at room temperature. The denatured DNA was diluted to 15 pM and spiked with 25% of the Illumina PhiX control DNA prior to loading the sequencer. Illumina paired-end sequencing was performed on the MiSeq platform, using a 500-cycle version 2 reagent kit. Illumina MiSeq paired-end reads were aligned to human reference genome Hg19 with bowtie2 and matching sequences discarded (60, 61). As previously described, the remaining nonhuman paired-end sequences were sorted by sample via barcodes in the paired reads with a python script (58). The sorted paired reads were assembled using phrap (62, 63). Pairs that did not assemble were discarded. Assembled sequence ends were trimmed over a moving window of 5 nucleotides until average quality met or exceeded 20. Trimmed sequences with more than one ambiguity or shorter than 200 nucleotides were discarded. Potential chimeras identified with Uchime (usearch6.0.203_i86linux32) (64) using the Schloss (65) Silva reference sequences were removed from subsequent analyses. Assembled sequences were aligned and classified with SINA (1.3.0-r23838) (66) using the 418,497 bacterial sequences in Silva 115NR99 (67) as reference configured to yield the Silva taxonomy. Operational taxonomic units (OTUs) were produced by clustering sequences with identical taxonomic assignments. This process generated 1,305,615 sequences for 43 samples (average sequence length: 315 nt; average sample size: 30,363 sequences/sample; range 679–166,133). The median Goods coverage score was 91.8% or higher at the rarefaction point of 679. The software package Explicet (v2.10.5, http://www.explicet.org) (68) was used for display, analysis; rarefied values for median Good’s coverage and α diversity; and calculation of Morisita-Horn (MH) β diversity values.

### Primary airway epithelial cell ALI culture.

Cryopreserved passage 1 airway epithelial basal cells from matched baseline and 6 mo. donor samples were differentiated in ALI culture using previously published methods (35). Equivalent numbers of cells were seeded onto collagen I–coated Transwell filters (Corning), and allowed to proliferate to confluence in homemade proliferation medium supplemented with Y-27632 (ROCK inhibitor, 10 μM). Cells were cultured at ALI in a 1 to 1 mix of homemade differentiation medium and PneumaCult-ALI medium (STEMCELL Technologies) for 21 days. The mix of homemade and commercial media was used to support some baseline cultures that otherwise did not survive to complete differentiation in the less supportive homemade media. To model in vitro CFTR modulation, some cultures were treated with elexacaftor (VX-445, 3 μM), tezacaftor (VX-661, 3 μM), and ivacaftor (VX-770, 0.1 μM) (all Selleckchem) during the entirety of ALI differentiation (35). TEER was measured using an EVOM epithelial volt/ohm meter (World Precision Instruments). To assess epithelial junctions and cellular differentiation, cultures were fixed in 4% paraformaldehyde for 15 minutes at room temperature, permeabilized in 0.1% Triton X-100 in PBS (PBST) for 5 minutes, and then blocked in 10% normal horse serum in PBST for 1 hour. Primary antibodies (anti-FOXJ1, 1:500, catalog 14-9965-82, Thermo Fisher Scientific; anti-MUC5AC, 1:500, catalog MA5-12178, Thermo Fisher Scientific) were added overnight in blocking solution at 4°C. Alexa dye–conjugated secondary antibodies (Thermo Fisher Scientific) were added in blocking solution at room temperature. Cells were stained with Alexa Fluor 633–Phallodin (Thermo Fisher Scientific). Membranes were mounted in Mowiol mounting medium containing 2% *N*-propyl gallate (Sigma-Aldrich) and imaged with a Zeiss LSM 900 confocal microscope.

### Statistics.

Statistical analysis was carried out using GraphPad Prism software unless otherwise noted. Statistical tests are indicated in the figure legends. *P* values and adjusted *P* values of less than 0.05 were considered significant. Box-and-whisker plots consist of a box bound by the 25th percentile (bottom edge) and the 75th percentile (top edge) and a line through the box at the median, and whiskers extending to the minimum value (lower) and the maximum value (upper) in the dataset.

### Study approval.

Nasal brushes and clinical data were collected under protocol no. HS-3236 approved by the Institutional Review Board of National Jewish Health. All participants provided written informed consent.

### Data availability.

Data are available in the NCBI GEO under accession numbers GSE306517 and PRJNA1234118. Values for all data points shown in graphs are reported in the Supporting Data Values file.

## Author contributions

EKV carried out experiments, analyzed data, acquired funding, and wrote the manuscript; all authors edited the manuscript. AEG, SSN, DB, MS, EP, MAS, KBH, and MRM analyzed data. SY and JKH carried out experiments and analyzed data. AWMW collected clinical samples and data. SMH, DAL, DMB, and JLTC analyzed clinical data. DMB and JLTC acquired funding and supervised clinical sample and data collection.

## Funding support

- Cystic Fibrosis Foundation grants TAYLOR19A0 (to DMB and JTC) and VLADAR18GO (to EKV).
- Marshall and Margherite McComb Foundation.

## Supplementary Material

Supplemental data

Supplemental table 1

Supplemental table 2

Supplemental table 3

Supplemental table 4

Supplemental table 5

Supplemental table 6

Supplemental table 7

Supplemental table 8

Supplemental table 9

Supplemental table 10

Supplemental table 11

Supplemental table 12

Supporting data values

## Figures and Tables

**Table 3:**
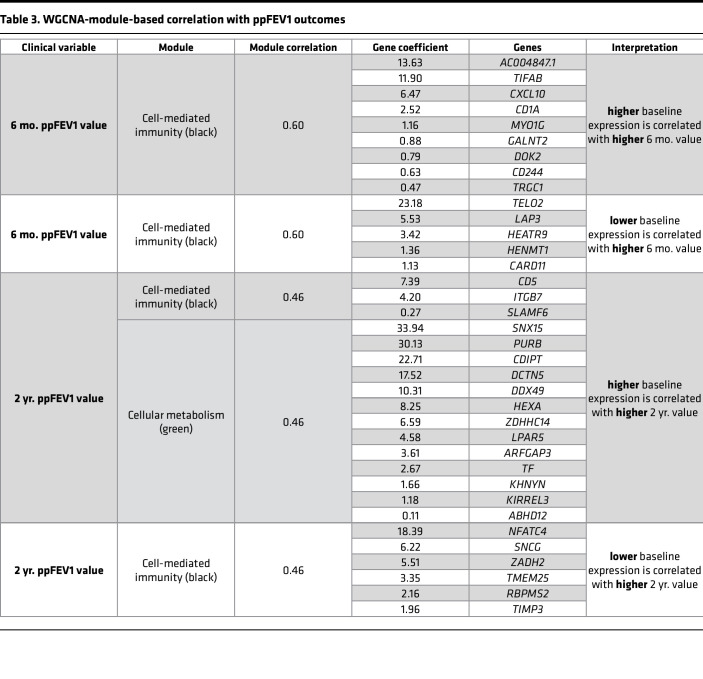
WGCNA-module-based correlation with ppFEV1 outcomes

**Table 2:**
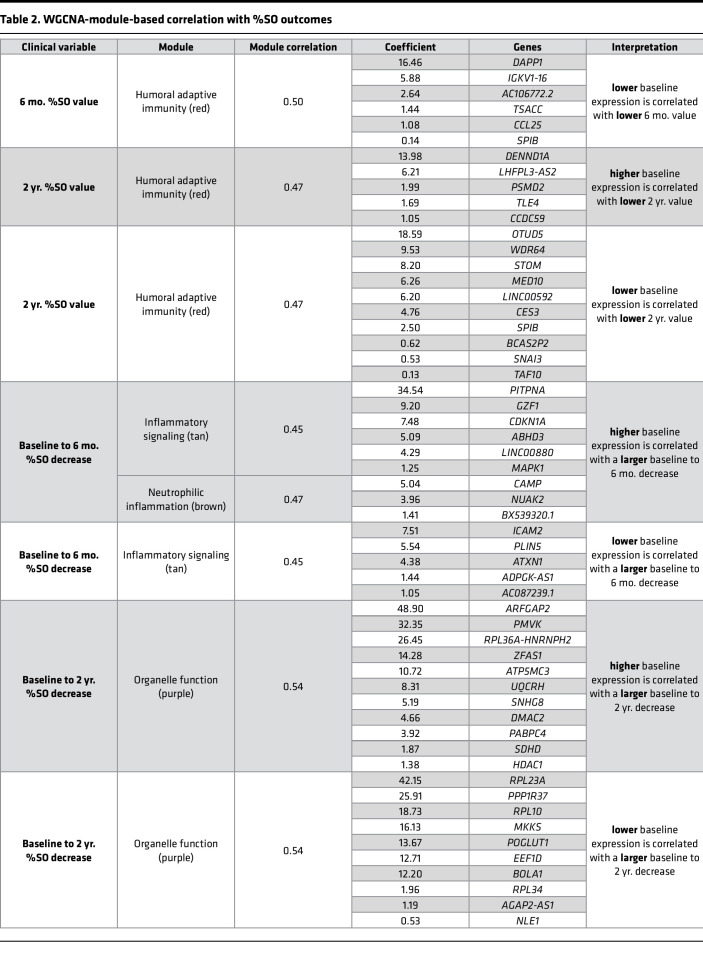
WGCNA-module-based correlation with %SO outcomes

**Table 1:**
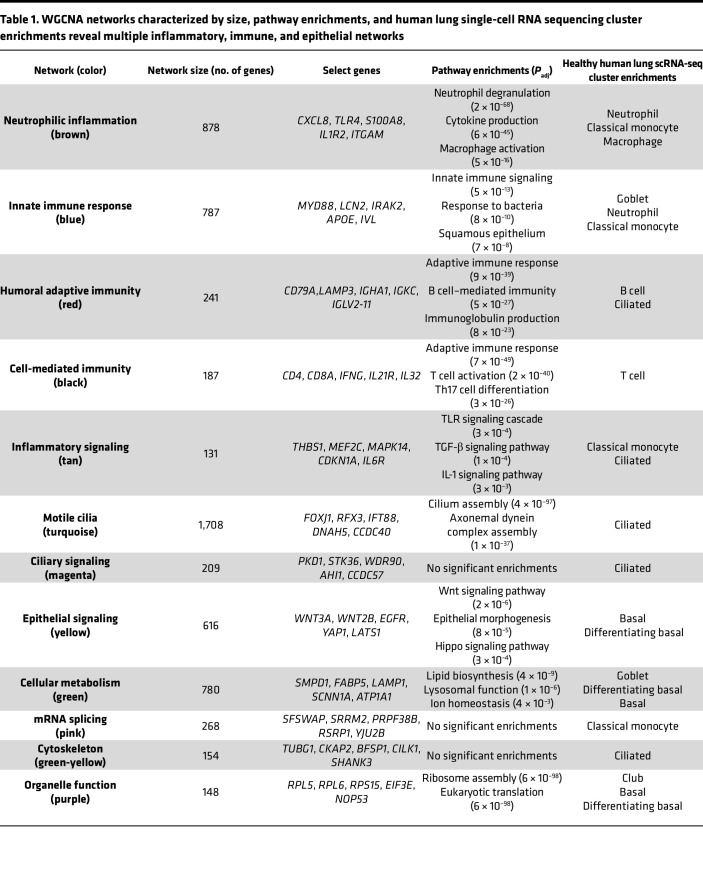
WGCNA networks characterized by size, pathway enrichments, and human lung single-cell RNA sequencing cluster enrichments reveal multiple inflammatory, immune, and epithelial networks

## Version 1 11/10/2025

Electronic publication
