# Supplementary material for: Transcriptomic and functional responses of the cystic fibrosis airway epithelium to CFTR modulator therapy
Source: JCI Insight. 2025 Nov 10;10(21):e196018. doi: 10.1172/jci.insight.196018 (PMC12643517; doi:10.1172/jci.insight.196018)
Supplement: Supplemental data [file jciinsight-10-196018-s208.pdf]

Supplemental figures and legends

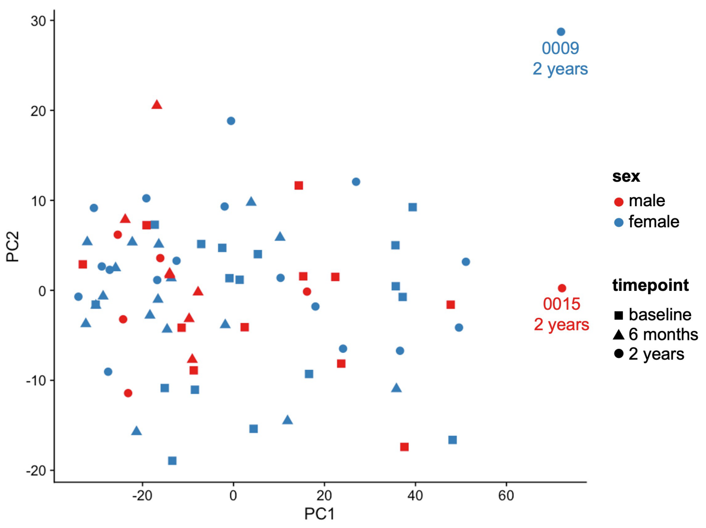

Supplemental Figure S1 (Vladar)

Supplemental Figure S1 Principal component analysis (PCA) identifies potential transcriptional outliers. PCA plot of study samples after sex regression with the two potential 2 yr. outliers indicated.

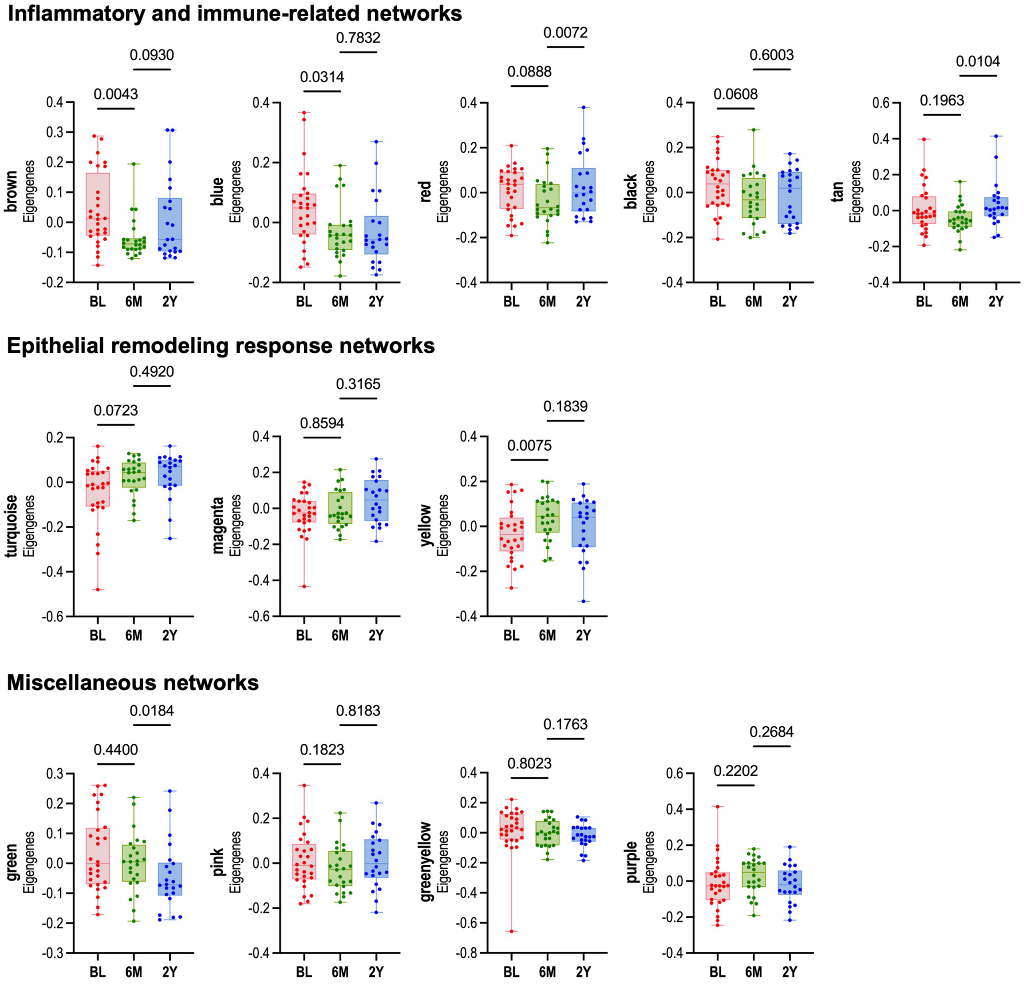

Supplemental Figure S2 (Vladar)

Supplemental Figure S2 WGCNA module changes over time in response to ETI treatment. Graphs per module show box and whisker plots with 2-way ANOVA.

A

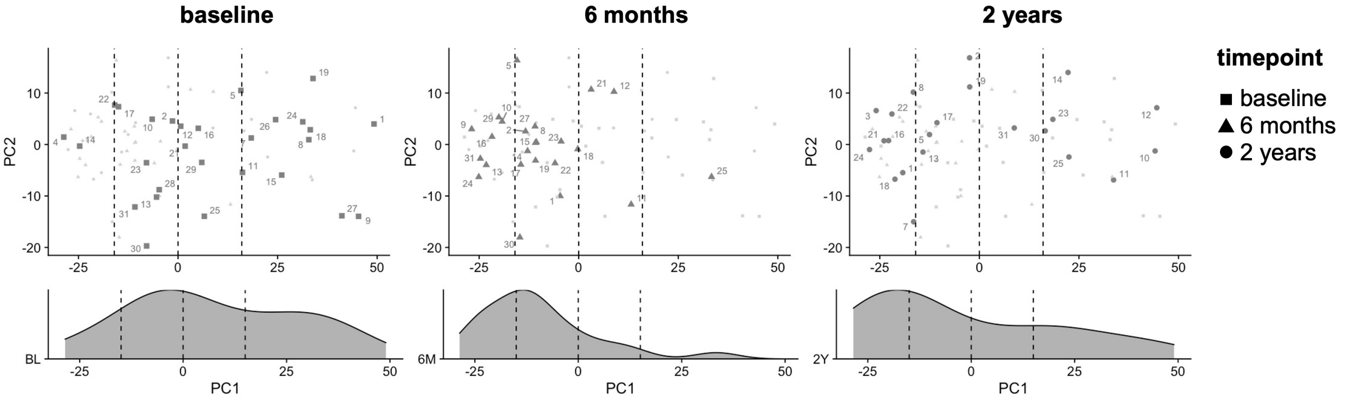

B

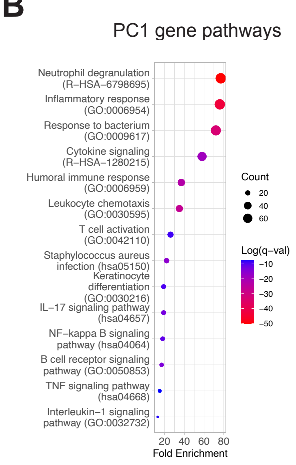

Supplemental Figure S3 (Vladar)

Supplemental Figure S3 Decreased inflammatory gene expression reverses after long term ETI treatment. A. PCA plots of study samples separated by timepoint. Graphs below indicate enrichment of samples along principal component 1 (PC1). **B.** Pathway analysis of genes that define PC1 reveals a strong enrichment in inflammation and immune function related processes.

A Healthy human lung reference data

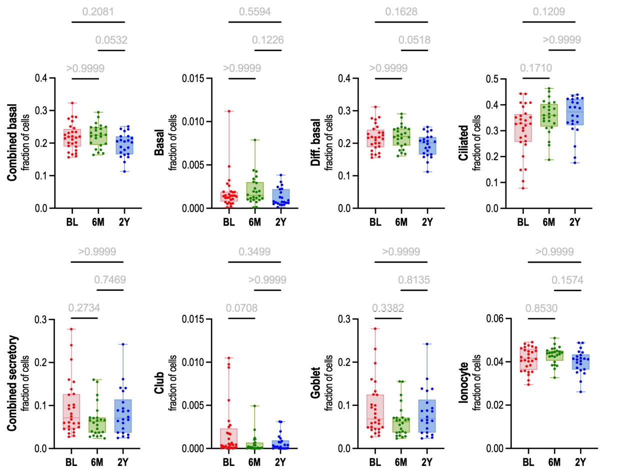

B Healthy human lung reference data

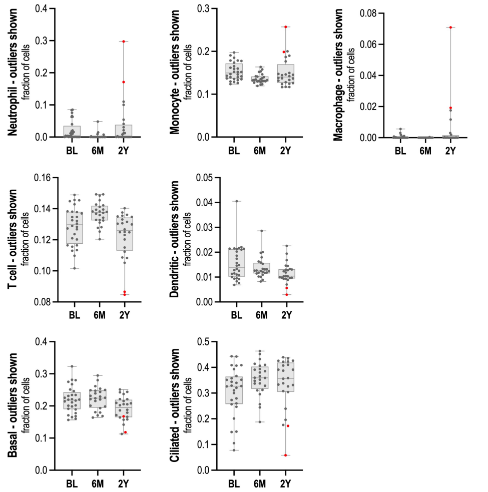

Supplemental Figure S4 (Vladar)

Supplemental Figure S4 Epithelial cell proportionality changes and 2 yr. transcriptional outlier samples. **A.** Computational deconvolution of epithelial cell type proportions based on a healthy human lung single cell RNA sequencing reference dataset indicates a trend towards an increase in ciliated cells and a decrease in basal stem cells over time. Graphs show box and whisker plots with Kruskal-Wallis test. **B.** 2 yr. outliers contain a very high proportion of neutrophils, monocytes, and macrophages, and low proportions of T cells, dendritic cells, and epithelial cells. Red dots indicate the two 2 yr. outliers.

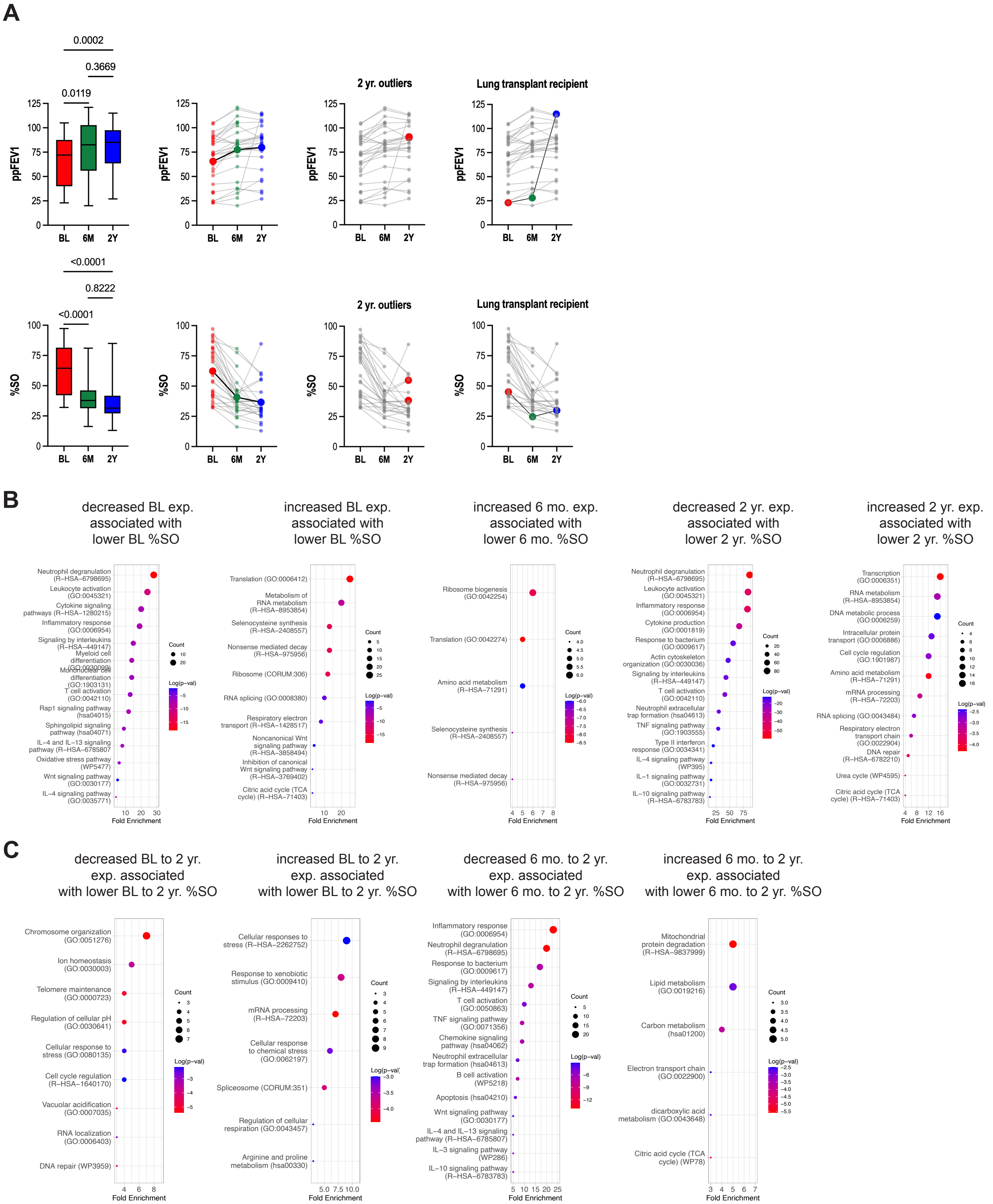

Supplemental Figure S5 (Vladar)

Supplemental Figure S5 Clinical outcomes for the cohort and per timepoint associations. A. Forced expiratory volume in one second percentage predicted (ppFEV1, top) and percent sinus opacification (%SO, bottom) show improvements between baseline and 6 mo. and no significant change from 6 mo. to 2 yr. Data include the two participants that correspond to the 2 yr. transcriptional outliers. Graphs show box and whisker plot with 2-way ANOVA test, left; individual datapoints throughout timecourse overlaid with per timepoint mean, center left; individual datapoints with the two 2 yr. outliers highlighted in red, center right; and individual datapoints with participant who received a lung transplant between 6 mo. and 2 yr. highlighted, right. **B.** Pathway enrichments for genes associated with lower SO% value at the indicated timepoints. **C.** Pathway enrichments for genes associated with lower SO% value at the indicated timeframes.

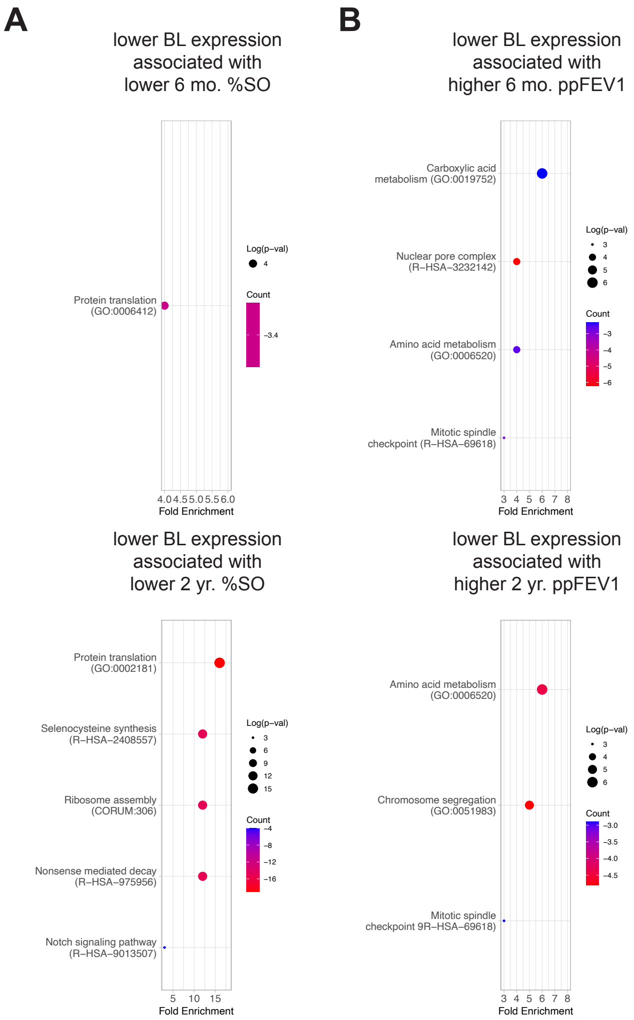

Supplemental Figure S6 (Vladar)

Supplemental Figure S6 Baseline gene expression association to clinical outcomes after ETI therapy. A. Pathway enrichments for genes whose decreased expression at baseline is associated with a greater decrease in %SO at 6 mo. (left) and 2 yr. (right). **B.** Pathway enrichments for genes whose decreased expression at baseline is associated with a greater increase in ppFEV1 at 6 mo. (left) and 2 yr. (right).

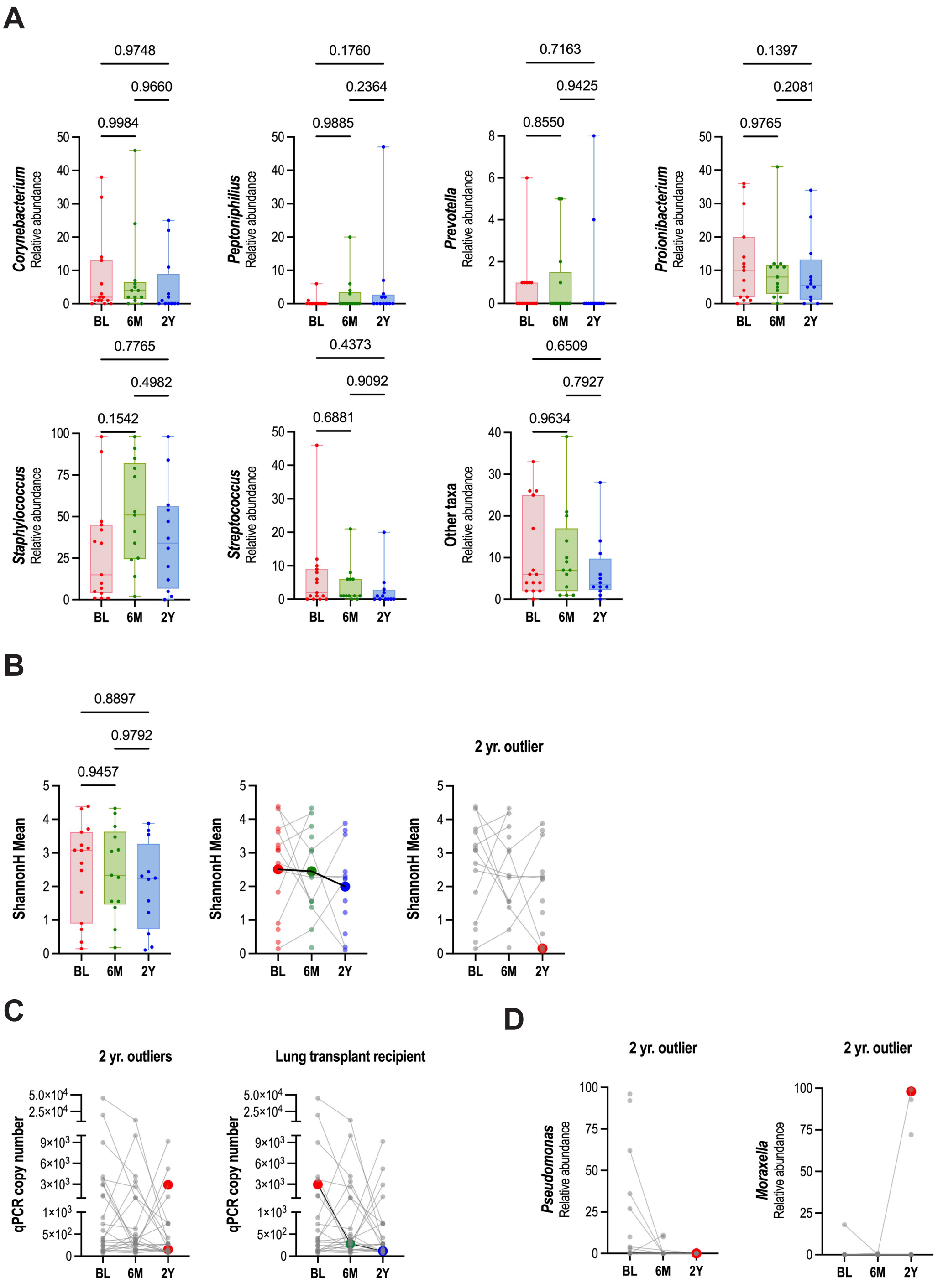

Supplemental Figure S7 (Vladar)

Supplemental Figure S7 Sinonasal microbiome changes in response to ETI therapy. A. Relative abundance among the top 25 sequenced taxa indicates no significant changes in select taxa of interest during the timecourse. Graphs show box and whisker plots with 2-way ANOVA test. **B.** Mean Shannon diversity index (H) indicates no significant changes in the top 25 most abundant taxa during the timecourse. Graphs show box and whisker plots with 2-way ANOVA test, left; individual values with mean highlighted, center; and individual values with 2 yr. outliers highlighted in red, right. **C.** 16S rRNA copy number values showing individual datapoints highlighting the two 2 yr. outliers, left; and the timecourse for participant who received a lung transplant between 6 mo. and 2 yr., right. **D.** *Pseudomonas* (left) and *Moraxella* (right) relative abundance graphs showing individual datapoints highlighting one of two 2 yr. outliers.

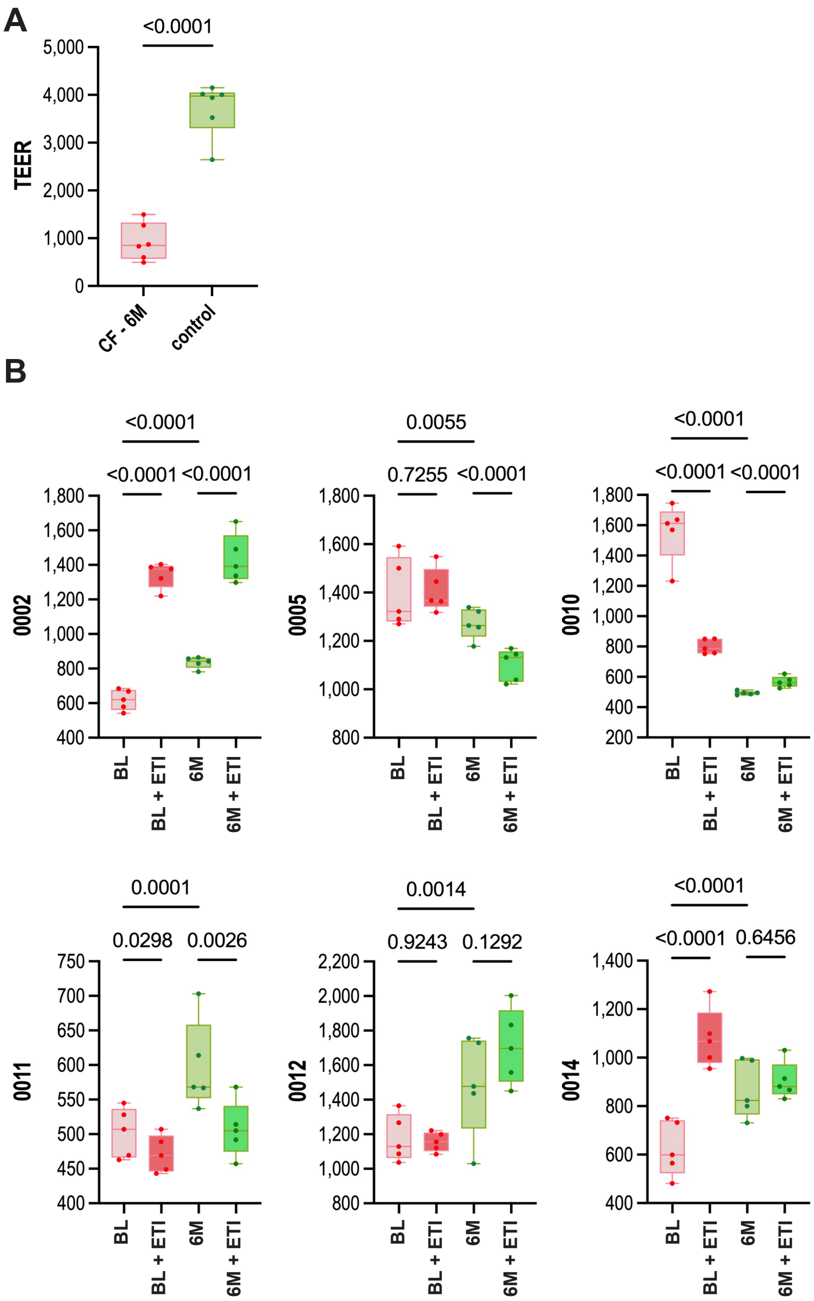

Supplemental Figure S8 (Vladar)

Supplemental Figure S8 In vitro ETI therapy has a variable effect on TEER in primary ALIs. A. TEER remains significantly lower in CF ALIs derived from 6 mo. samples compared to healthy control donor ALIs differentiated to the same stage of culture. Healthy controls show the average of three measurements from n=3 independent cultures. Graphs show box and whisker plot with two tailed t-test. **B.** Matched baseline and 6 mo. ALIs indicate increased barrier capacity at 6 mo. in most samples as measured by transepithelial electrical resistance (TEER, Ω·cm2). Treatment with ETI compounds during the entirety of differentiation had a variable effect. Individual sample graphs show the average of three measurements from n=5 independent cultures. Graphs show box and whisker plots with 2-way ANOVA test.
